# The current status of FLASH particle therapy: a systematic review

**DOI:** 10.1007/s13246-023-01266-z

**Published:** 2023-05-09

**Authors:** Jake Atkinson, Eva Bezak, Hien Le, Ivan Kempson

**Affiliations:** 1 Future Industries Institute, University of South Australia, Mawson Lakes, South Australia 5095 Australia; 2 Cancer Research Institute, University of South Australia, Adelaide, South Australia 5000 Australia; 3 Department of Physics, University of Adelaide, North Terrace, Adelaide, South Australia 5000 Australia; 4 Department of Radiation Oncology, Royal Adelaide Hospital, Adelaide, 5000 Australia

**Keywords:** FLASH radiotherapy, Ultra-high dose rate, Proton therapy, Carbon therapy, Normal tissue sparing, Biological mechanisms, Cancer treatment

## Abstract

Particle therapies are becoming increasingly available clinically due to their beneficial energy deposition profile, sparing healthy tissues. This may be further promoted with ultra-high dose rates, termed FLASH. This review comprehensively summarises current knowledge based on studies relevant to proton- and carbon-FLASH therapy. As electron-FLASH literature presents important radiobiological findings that form the basis of proton and carbon-based FLASH studies, a summary of key electron-FLASH papers is also included. Preclinical data suggest three key mechanisms by which proton and carbon-FLASH are able to reduce normal tissue toxicities compared to conventional dose rates, with equipotent, or enhanced, tumour kill efficacy. However, a degree of caution is needed in clinically translating these findings as: most studies use transmission and do not conform the Bragg peak to tumour volume; mechanistic understanding is still in its infancy; stringent verification of dosimetry is rarely provided; biological assays are prone to limitations which need greater acknowledgement.

## Introduction

It is recommended that X-ray radiation therapy (RT) be included in the treatment of 50% of all cancer patients in developed countries worldwide, making it one of the most common modes of treatment available currently [1]. Accounting for only 5% of total cancer therapy costs, as well as being a non-invasive technique, it is appealing in both its low economic burden and practicality [2]. Although there are significant advantages to utilising conventional X-ray RT, this modality has the potential to cause detrimental off-target effects, inherent in the physics involved. Due to exponential absorbed dose by an X-ray beam, normal tissues are exposed to radiation prior to entering, and upon exiting, a tumour site. This may produce severe, negative side effects in patients if highly sensitive tissues lie within the radiation track [3]. For instance, the potential tissue complications that can result from lung irradiation include early onset pneumonitis, late onset fibrosis (occurring in 5–20% of patients), and risks of heart or spine irradiation depending upon tumour location [4]. The risks that RT complications present are amplified in paediatric patients. Brain irradiation presents risk of cognitive decline and growth hormone deficiencies in paediatric patients compared to their healthy peers [5, 6]. Second malignancy induction is also a prominent issue in the paediatric and young adult population due to their longer survivorship period [7]. Therefore, limiting the degree of normal tissue damage, and consequent accumulation of toxicity, is pivotal in preventing late effects from occurring. Despite success of RT to date, there is still demand for new and innovative treatment modalities to mitigate radiation damage to peripheral normal tissues, with advances being essential for improving both the rate of cancer survival and the quality of life of patients after treatment. Identifying irradiation techniques which broaden the therapeutic window by minimising normal tissue damage will allow for a lower incidence of negative side effects in patients, and even allow for greater radiation doses to be delivered to improve the probability of tumour control [8].

Particle therapy is one such modality that is continually being developed and currently being employed clinically for this reason. It employs the use of high Linear Energy Transfer (LET) particles to irradiate treatment volumes with minimal exit dosage compared to X-ray photons (low LET), reducing irradiation of surrounding healthy tissues. X-rays deliver their maximum dose at the point of electronic equilibrium and then attenuate exponentially as they pass through the remaining tissue, prior to and upon exiting the tumour. Conversely high LET particles, including protons or carbon ions, have low energy deposition upon entry and maximum deposition at the Bragg Peak towards the end of their track. Particle therapy centres are now employing intensity modulated and pencil beam scanning techniques, which target tumours in layers of irradiation spots at differing doses, depths, and positions [9]. The benefits of such treatment include better dose conformation, minimisation of damage to healthy tissues and an increased total dose that can be administered [10]. Use of high LET particles, particularly carbon ions, allows for efficient treatment of radioresistant tumours localised within critical organs where X-ray or proton beam therapies would be ineffective.

The resurgence of studies involving irradiation of tissue at ultra-high dose rates, also known as FLASH radiation therapy (FLASH-RT), alongside development of proton and carbon ion therapies, are steps towards further improving the therapeutic efficacy of RT. FLASH-RT typically delivers absorbed dose rates greater than 40 Gy/s to irradiate tumours in very small timeframes (< 1 s), as opposed to conventional dose rates (CONV, < 0.1 Gy/s) which are normally fractionated and delivered over the course of minutes [11]. There is some evidence that the utilisation of FLASH technique, even with photon or electron beams, results in a radioprotective effect in normal tissues, which reduces toxicity compared to CONV. Multiple mechanisms have been hypothesised to explain this phenomenon, including rapid intracellular oxygen depletion preventing indirect DNA damage via reactive oxygen species (ROS) [12], alterations to the nature of DNA damage and DNA repair pathways, and immune response modulation [13]. However, the radiobiological processes by which these mechanisms occur during FLASH-RT are not well understood. All these factors are thought to contribute towards the unique normal tissue sparing effect of FLASH-RT, which has been previously demonstrated in vivo for photons, electrons, and heavy ions.

Incorporating the differential tissue response of the FLASH concept with the normal tissue sparing of particle therapy may prove to be beneficial when utilised in combination. Taking advantage of proton or carbon ion beam’s characteristics - particularly their minimal exit dose - alongside the normal tissue sparing properties afforded by FLASH, there is the potential to synergistically minimise healthy tissue toxicities. Although there are multiple studies outlining the biological outcomes of the FLASH effect with electrons and X-ray photons [14–16], few studies have explored the implications of using proton or carbon ions at ultra-high dose rates in vitro and in vivo. This systematic review summarises available literature detailing FLASH-RT’s discovery, its implementation in electron, proton and carbon ion-based studies, as well as discussion of conflicting data concerning the various hypotheses that form the basis of these studies. Overviews of theoretical, in vitro, and in vivo particle beam studies relevant to ultra-high dose rates, and their effect upon normal and cancer cell biology, are the primary focus with the aim of understanding current state of knowledge on mechanistic benefits that could further improve particle therapy outcomes in the future. From literature captured by the systematic search strategy, this review provides: a brief history of electron FLASH experiments; its recent resurgence in the literature in terms of in vitro, in vivo, and even clinical application; and the current state of research into FLASH delivery of protons and carbon ions.

## Methodology

### Systematic review process

To produce an accurate overview of the biological mechanisms of the FLASH effect, a systematic review of all literature pertaining specifically to particle-FLASH (excluding electrons) has been compiled. Since electron-FLASH publications were amongst the most prevalent throughout initial FLASH searches, the key electron-FLASH studies were included in a non-systematic manner in this manuscript to provide background and context to the particle-FLASH studies identified throughout the systematic process. The progression of the electron-FLASH field has been essential in providing preliminary data that will aid in translation into the particle-FLASH field.

The inclusion criteria for literature within review include:

- Scientific articles. Conference abstracts and reviews were excluded.
- Findings only pertaining to the radiobiological effect of ultra-high dose irradiation on normal and cancerous cellular function and response. Technical developments pertaining to proton and carbon-ion delivery were excluded.
- *In silico*, in vitro, in vivo, and clinical studies.
- Studies utilising ultra-high dose rates, defined as an average dose rate of greater or equal to 40 Gy/s.
- Articles published between the years 2009–2022.
- Language restricted to English (translations also accepted).

In completing this process systematically, multiple search criteria were utilised with increasing degrees of complexity (additional search terms, higher search specificity). Table 1 below outlines the set of search criteria used to identify proton and carbon-FLASH literature performed on the 8th December, 2022.

**Table 1:** Search strategy for proton- and carbon-FLASH articles, utilising an expanded set of FLASH, proton, and carbon therapy search terms. A total of 62 experimental studies were yielded from Scopus and Medline searches after duplicates were removed

Search #	Search Terms	Scopus	Medline
1	“FLASH-RT” OR “FLASH radiation therapy” OR “FLASH radiotherapy*” OR “FLASH effect*” OR “FLASH irradiation” OR “Ultra-high dose rate*”	662	396
2	“Proton therapy” OR “Proton beam therapy” OR “Proton radiation therapy” OR “Proton radiotherapy” OR “Proton beam irradiation”	12,562	8,447
3	“Heavy ion radiotherapy” OR “Heavy ion radiation therapy” OR “Carbon ion therapy” OR “Carbon ion irradiation” OR “Carbon ion radiotherapy” OR “Carbon ion radiation therapy” OR “Carbon beam therapy”	2,468	2,090
4	FLASH **AND** (Proton therapy **OR** Heavy ion/Carbon therapy) (#1 AND (#2 OR #3))	78	37

**Fig. 1:**
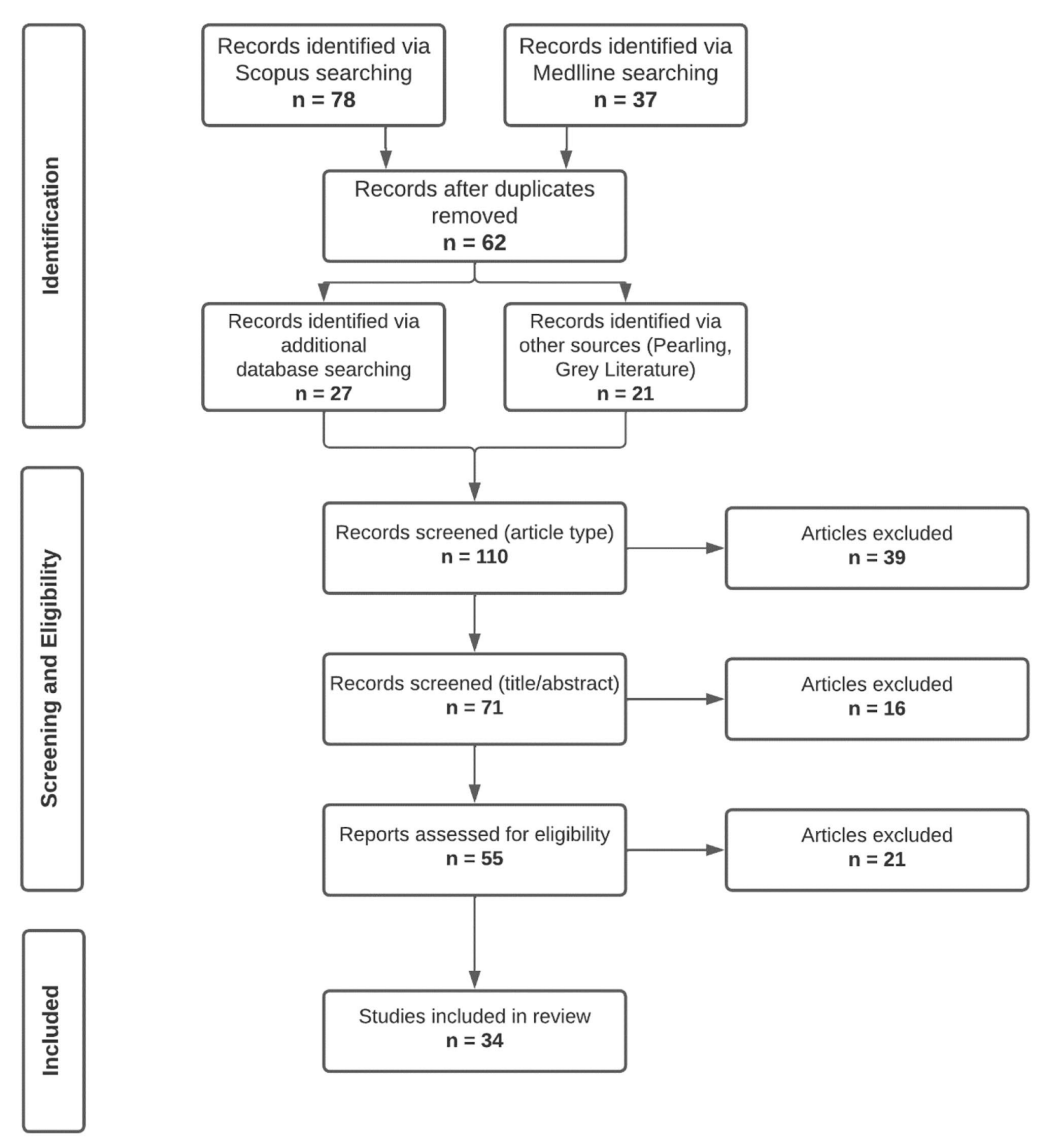
PRISMA flowchart summary of the search strategy employed in this systematic review. Search terms were filtered through either Scopus or Medline databases and then excluded based upon their relevance to the radiobiological effect of FLASH and ultra-high dose rates. Additional terms were then added from keywords or terminology discovered whilst reading through relevant literature and searches repeated in both databases. Sources were then reviewed by co-authors of this review and categorised. 34 proton- and carbon-FLASH studies are incorporated into the final review

Studies from search #4 of Table 1 were added to the studies found in the initial literature searches. In addition to utilising Scopus and Medline to compile resources, other articles were found via selecting relevant citations from systematically identified literature (pearling). Finally, additional sources were identified via grey literature searching with Google Scholar. This resulted in the culmination of 110 publications, which were then screened by IK to validate their relevance, followed by EB to resolve any disagreement in relevance between IK and JA. The final number of systematically identified proton and carbon therapy papers was 34. A diagrammatic summary of the PRISMA search strategy utilised for this systematic review is displayed in Fig. 1. PRISMA guidelines were followed in the literature identification and exclusion process [17]. Relevant electron-FLASH papers identified in a non-systematic manner were summarised in table format (Table 2), with systematically identified particle-FLASH literature being summarised in the same manner (Table 3), listing year of publication, particle type, irradiation source and mode, model (e.g., *in vitro, in vivo)*, particle energy (MeV), doses (Gy), delivery time, pulse/burst count and rate, FLASH average and instantaneous dose rate per fraction (Gy/s), comparative low or CONV dose rate, (Gy/s) and key experimental conclusions. Throughout the text, average dose rates are discussed unless specified.

## History of FLASH radiotherapy

Although the first publications that cite the use of radiation at ultra-high dose rates did not initially quote the term ‘FLASH’ irradiation, coined by Favaudon et al. in 2014 [18], interest in the field was motivated earlier by exploring the relationship between radiation dose rate and biological response. In 1958, Kirby-Smith and Dolphin published one of the first papers that suggests a dose rate dependent effect on DNA lesion formation, showing that in a *Tradescantia* (spiderwort plant) model, the total number of chromosome aberrations was reduced when electrons were administered at higher dose rates of up to 4 × 10^6^ Gy/s [19]. The effect of cellular oxygenation on cellular radiosensitivity was also topical, and in 1959 Dewey and Boag presented a radiobiological relationship between dose rate, cellular oxygenation, and cellular survival. They aimed to observe the effect of dose rate on the surviving fraction of *Serratia marcescens* bacteria, whilst modifying both total irradiation dose and the concentration of dissolved oxygen. Bacteria were irradiated in the presence of both CONV X-rays (0.1 Gy/s) and ultra-high dose rate electrons (5 × 10^7^ – 10 × 10^8^ Gy/s) with total absorbed doses ranging between 0 and 200 Gy, and also varying oxygen concentrations (100% O_2_, 1% O_2_ in N_2_ and 100% N_2_). The proportion of surviving bacteria was higher at the ultra-high dose rate irradiation, which was comparable to the radioprotection afforded under anaerobic conditions at the conventional dose rate [20]. This was theorised to be a result of the initial absorbed dose of radiation ‘consuming’ the oxygen dissolved within the irradiated bacterium, thus preventing the production of harmful reactive oxygen species that would usually result in cytotoxic DNA damage and apoptosis. Also lending to the theory, the authors suggested that the irradiation timeframe was so minute, that even if dissolved oxygen was not completely removed from the extracellular environment, it would not be able to re-enter the cell rapidly enough for the radiation pulse to dissociate it. This theory, termed the ‘oxygen effect’, would consequently form part of the hypothesis behind the healthy tissue sparing effects of FLASH irradiation. Similar publications around this time held interest in the relationship between dose rate and ROS concentration [21], and if there were analogous FLASH effects in *E. coli* [22].

Further work looking into the dose rate effect in the survival of mammalian cells was conducted by C.D. Town in 1967. Using 3.5 × 10^7^ Gy/s electrons, experiments were conducted comparing HeLa cell survival as a function of radiation dose, between single and double pulses of radiation. When cells were exposed to doses higher than 10 Gy, there was an apparent sparing of tissues; cells treated with doses administered in a single burst of radiation had a higher survival fraction compared to irradiation over two pulses. To validate whether this tissue sparing was related to the oxygen effect, an additional test, wherein cell suspensions were exposed to either nitrogen or air, was performed. Single pulse irradiations under aerated conditions resulted in similar cell survival curves to that of irradiation under anaerobic conditions (at doses exceeding 10 Gy) [23]. These findings, similar to Dewey and Boag’s *S. marcescens* experiments, supported that the ‘oxygen effect’ is not limited to bacterial cells; indeed, there was sufficient evidence alluding to its role in mammalian cell protection. In the present day, the definition of the ‘FLASH effect’ phenomenon has formed as the sparing effect ultra-high dose rates have upon healthy, but not cancerous, tissues in vivo, whereas this was not the primary aim of these initial studies. Nevertheless, they highlight the discovery of dose-rate dependent radiobiological responses, and paved the way for FLASH’s re-emergence as a potential cutting-edge treatment modality decades later.

## Electron FLASH

### Resurgence of FLASH

Studies concerning ultra-high dose rate irradiation faded into obscurity for some 30 years until its sudden resurgence in the 2010s. Although in previous publications in vitro experiments predominated, more recent work aimed to identify how ultra-high dose rates benefited normal tissues *in vivo.* Radiation-induced pulmonary fibrosis is a side effect [24] with the potential for treatment-related death following external beam irradiation [25]. As such, multiple in vivo studies have been performed to determine whether utilising ultra-high dose rates may elicit a greater differential response between normal and tumour tissue damage compared to current clinical dose rates. A summary of the experimental results of these electron-FLASH papers is presented in Table 2 below.

**Table 2:** Summary table of key electron-FLASH literature identified non-systematically. For FLASH irradiations, delivery time, pulse count and rate, average dose rate per fraction, instantaneous dose rate (within one pulse), and comparative CONV dose rate are provided. Data listed in bold have been calculated from information provided in the text

Ref.	Year	Model	Energy (MeV)	Dose (Gy)	Delivery Time (s)	Pulse Count	Pulse Rate (Hz)	FLASH Average Dose Rate (Gy/s)	FLASH Instantaneous Dose Rate (Gy/s)	Low or CONV Dose Rate (Gy/s)	Outcome
[26]	1971	Random bred TO strain mice	7	**8–40**	NM*	NM*	400	**16–83**	**> 2 × 10** ^**4**^	**1, 2.5**	The LD_50_ of mice 4–5 days post irradiation was increased for whole body irradiation at dose rates of 16–83 Gy/s compared to 1 Gy/s when breathing oxygen. N_2_ breathing mice had comparable LD_50_ to the oxygen breathing mice at high dose rates, with the prediction that a reduction in oxygen tension was responsible.
[27]	1974	Rat, hind leg	7	**20–80**	< 0.5	NM*	NM*	**66.7–83.3**	NM*	**0.03**	Decreased level of skin damage with increasing dose rate under aerobic conditions. Break away response at 20 Gy, where skin irradiation of hindlegs was comparable to anoxic conditions (reduced radiosensitivity). Suggests oxygen depletion is responsible for radioprotection at high dose rates.
[28]	1977	Human lymphocytes, blood taken from adult male donor	15	**0.44–7.64**	1 × 10^− 6^	1	Single - Pulse	**5.3 × 10** ^**5**^ **– 7.64 × 10** ^**6**^	**5.3 × 10** ^**5**^ **– 7.64 × 10** ^**6**^	**1**	Absence of dose-dependent effect on chromosomal aberration yield for 0.44–7.64 Gy doses in vitro. Dose-response relationship using electrons is comparable to previous X-ray and gamma-ray irradiation studies.
[18]	2014	C57BL/6J mice, bilateral thorax irradiation, HBCx-12a, HEp-2 tumour xenografts, TC-1 Luc^+^ orthotopic lung carcinoma	4.5	7.5, 17, 30	> 0.1	4–6	100–500	60	1 × 10^6^	0.03	No complications were observed in mice after thoracic irradiation at FLASH dose rates below 23 Gy. Pulmonary fibrosis, TGFβ cascade induced in CONV irradiation at 17 Gy, 30 Gy FLASH required to induce same effects. FLASH also prevented acute apoptosis in bronchial epithelia, smooth muscle, and vasculature. The tumour killing efficacy of FLASH was comparable to CONV irradiations at equivalent doses.
[29]	2015	Human squamous cell carcinoma: FaDu and SKX.	20	1–10	≤ 1 × 10^− 3^	**~ 13,000**	1.3 × 10^7^	**9 × 10** ^**4**^	**1.67 × 10** ^**8**^	**0.006, 0.067**	No appreciable difference between different ELBE** beam pulse regimes and resulting mean dose rates, including clonogenic survival response and the number of residual γH2AX or 53BP1 foci present 24 h post irradiation.
[30]	2016	Normal tissue mammary gland breast epithelium 184A1, normal neonatal foreskin-derived dermal fibroblasts HDF	20	4, 8	≤ 1 × 10^− 3^	**~ 13,000**	1.3 × 10^7^	**9 × 10** ^**4**^	**1.67 × 10** ^**8**^	**0.006, 0.067**	No radiobiological difference in clonogenic survival or DNA DSB*** between cells irradiated at CONV or FLASH dose rates for 184A1 and HDF cell lines. Additionally, there appeared to be no discernible difference in γH2AX or 53BP1 foci formation between either dose rate used 24 h post irradiation.
[31]	2017	C57BL/6J mice, whole brain irradiation	6	10	1.8 × 10^− 6^ – 0.3	1–10	100	60–5.6 × 10^6^	1.9 × 10^5^ – 5.6 × 10^6^	0.1–30	FLASH whole brain irradiations appeared to spare hippocampal neurogenesis, evidenced by increased BrdU positive neural clusters in 10 Gy FLASH irradiated mice compared to CONV. No substantial difference in Recognition Ratio between FLASH (75.9 ± 4.0%) and non-irradiated (78.3 ± 2.6%) mice (CONV = 53.0 ± 1.7%). Loss of a neuroprotective effect was observed at dose rates < 30 Gy, however a memory preserving effect was regained at dose rates > 100 Gy/s.
[32]	2017	C57BL/6 mice, total abdominal irradiation	20	10–22	NM*	NM*	NM*	70, 210	NM*	0.05	Mice exposed to 13–19 Gy whole abdomen radiation exposure exhibited a survival rate of 29% in the CONV group, and 90% survival for those administered FLASH 20 days post-irradiation. The LD_50_ were 14.7 Gy for CONV irradiation and 17.5 Gy for FLASH.
[33]	2019	Pig model, skin irradiations, cat patients, T2/T3N0M0 squamous cell carcinoma (nasal)	4.5, 6	25–41	0.09–0.1	9–10	100	> 277	**0.5 × 10** ^**6**^ **– 1.8 × 10** ^**6**^	**0.083**	Mini Pigs: FLASH irradiations of pig skin had greatly reduced tissue toxicities compared to CONV, including preservation of hair follicles and CD34^+^ cells, and the prevention of erythema, moist desquamation and fibronecrosis. Cats: FLASH patients exhibited permanent depilation, mild mucositis/dermatitis, and no late toxicities. Progression-free survival 16 months post-treatment was 84% versus 50–60% 12 months post treatment at CONV dose rates.
[34]	2019	75-year-old human male, CD30 + T-cell cutaneous lymphoma	5.6	15	0.09	10	100	**158**	**1.5 × 10** ^**6**^	NM*	Epidermal thickness and basal membrane unaltered. Grade 1 epithelitis and transient grade 1 oedema observed 3 weeks post irradiation. Complete and rapid tumour response.
[35]	2019	C57BL/6 mice, BALB/c mice, murine pancreatic cancer cell lines KPC and Panc02, human peripheral blood mononuclear cells (PBMCs)	20	0–16	NM*	NM*	180	32.6, 38.8	NM*	0.1	FLASH induces higher rates of clonogenic cell death and apoptosis in pancreatic cancer cell lines. Comparable lymphocyte killing capacity to CONV. FLASH did not preserve immune cells irradiated via either cardiac or splenic tissues, induced higher degree of gastrointestinal toxicity than CONV in vivo.
[36]	2019	C57BL/6J mice, whole brain irradiation,	6	10, 12, 14	1.8 × 10^− 6^	1	100	**5.6 × 10** ^**6**^ **– 7.8 × 10** ^**6**^	**5.6 × 10** ^**6**^ **– 7.8 × 10** ^**6**^	**0.09–0.16**	Neurocognitive dysfunction was not induced in FLASH irradiated mice, evidenced by novel object recognition, object in place, temporal order, elevated plus maze, light-dark box and fear memory tests being comparable to non-irradiated controls. Increasing pO_2_ levels in the brain with carbogen breathing resulted in a loss of neurocognitive sparing during FLASH. FLASH also had lower levels of gliosis markers compared to CONV. FLASH irradiated zebrafish embryos exhibited a lower degree of morphological changes versus CONV.
		Zebrafish embryos		8	1.49 × 10^− 6^	1	200	**5.4 × 10** ^**6**^	**5.4 × 10** ^**6**^	**0.06**	
[37]	2019	C57BL6/J mice, whole brain irradiation	16, 20	30	0.1–0.16	18	106, 180	200, 300	8.75 × 10^5^	0.13	Neurocognitive deficits were not observed in mice 10-weeks post irradiation. FLASH mice exhibited preservation of hippocampal dendritic spines, reduced microglial inflammation and reduced expression of inflammation mediator cytokines. Novel object recognition and object location were not impaired in FLASH mice.
[38]	2020	C57BL/6J mice, whole brain irradiation	6	8	1.8 × 10^6^	1	100	4.4 × 10^6^	4.4 × 10^6^	0.1	FLASH and low dose rate mice lacked memory recall 2 months-post treatment. At 4-months, FLASH mice regained memory recall/update ability. FLASH irradiation also preserved novel object recognition and social interaction capabilities similar to control mice.
[39]	2020	C57BL/6J mice, whole brain irradiation	6	10	1.8 × 10^− 6^	1	100	5.6 × 10^6^	5.6 × 10^6^	0.1	In FLASH irradiated mice, the expression of astrogliosis factors was reduced compared to CONV irradiation, including toll-like receptor 4 (TLR4) and glial fibrillary acidic protein (GFAP). However, expression of complements C1q and C3 were elevated under both FLASH and CONV conditions.
[40]	2020	Prostate cancer cell line DU145	10	0–25	**0.005–0.03**	**1–6**	200	600	NM*	**0.23**	Negligible difference between FLASH and CONV under normoxic conditions, however a higher proportion of cells survived under hypoxic conditions. Under different relative partial O_2_ pressures, FLASH had higher surviving fractions of cells for 1.6%, 2.7% and 4.4% pO_2_.
[41]	2020	C57BL/6J mice, whole brain irradiation	6	10, 25	0.01, 1.8 × 10^− 6^	1, 2	100	2.5 × 10^3^, 5.6 × 10^6^	5.6 × 10^6^, 6.9 × 10^6^	0.09	FLASH reduced apoptosis in neurogenic brain regions 1-day post irradiation. Vascular dilation and downregulation of tight junction proteins occurred 1–4 weeks after CONV treatment but was minimal in FLASH mice, thereby preserving vasculature integrity in the brain.
[11]	2020	C57BL/6 mice, skin irradiation	16	10, 40	**0.056–0.22**	**5–20**	90	180	4.0 × 10^5^	0.075	FLASH irradiations of mice skin resulted in a lower incidence of ulceration in combination with reduced skin toxicities compared to CONV. 30 and 40 Gy hemithoracic irradiations also resulted in reduced mortality in FLASH irradiated mice (median survival > 180 days, both doses) versus CONV (median survival = 100 days at 30 Gy, 52 days at 40 Gy).
[42]	2020	Monte-Carlo simulation, EGSnrc, paediatric whole brain irradiation simulation	40	3	NM*	NM*	1000	115	NM*	NM*	In the treatment of paediatric brain tumours, there is theoretically less homogeneity in dose distribution of 40 MeV FLASH dose rate electrons compared to 6 MeV CONV photons due to electron scattering.
[43]	2020	C57BL/6 mice, total abdominal irradiation, ID8 ovarian cancer cells	16	12, 14, 16	**0.056–0.074**	**6, 7, 8**	108	216	NM*	0.079	Sparing of lethal intestinal injury and preservation of intestinal function was observed in normal and tumour-bearing mice. FLASH tumour killing effectiveness matched CONV in the treatment of ID8 ovarian cancer.
[44]	2020	C57BL/6J wild type and Terc^−/−^ mice, bilateral thorax irradiation, human lung fibroblast MRC5 and IMR-90 cells, human lung epithelial carcinoma A-549, pulmonary bronchial epithelial cells	4.5	2, 4, 5.2, 17	0.033–0.11	5–11	100–150	135–600	8 × 10^5^ – 3.2 × 10^6^	0.03	30 min post irradiation, significantly fewer 53BP1 foci in fibroblast cell lines, whereas γH2AX showed minimal variation compared to CONV dose rate. FLASH irradiated lung tissue exhibits fewer 53BP1 foci compared to CONV, and partially prevents the onset of fibrosis. Half the number SA-β-gal^+^ senescent clusters were produced under FLASH compared to CONV at 4 months post treatment.
[45]	2021	Human T-ALL M106 cell xenografts, hematopoietic stem cell progenitor cells, CD34^+^ cells, BRγc-/- mice	6	4	0.02	3	100	200	7.4 × 10^5^	0.072	Total body irradiation at FLASH dose rates controlled the propagation of T-ALL cases with similar genetic abnormalities. FLASH preserved hematopoietic stem cell progenitor and CD34^+^functionality.
[46]	2021	Bovine serum albumin solutions (5%), nude mice, MDA-MB-231 tumour model	10	20	**0.074–0.22**	**15–17**	120, 240, 360	50–300	2.14 × 10^5^, 2.38 × 10^5^	0.1	In vitro model: greater decrease in oxygen concentration observed in response to CONV vs. FLASH. High level O_2_: CONV = 3.8 mm Hg decrease in pO_2,_ FLASH = 3.1 mm Hg decrease in pO_2_. Low level O_2_: CONV = 4.2 mm Hg decrease, FLASH = 3.4 mm Hg FLASH decrease. In vivo model: FLASH depleted oxygen to a higher degree in normal tissues than tumour tissues, maintained at dose rates of 90, 180, 270 Gy/s and no statistically significant difference in O_2_ depleted between dose rates.
[47]	2021	NU_(Ico)_-Foxn1^nu^ female nude mice, brain irradiation, H454 orthotopic murine GBM model, U87 orthotopic murine GBM model	6	10, 14, 4 × 3.5, 2 × 7, 25, 3 × 10	1.8 × 10^6^, 0.01	1, 2	100	1.9 × 10^6^ – 7.8 × 10^6^	1.9 × 10^6^ – 7.8 × 10^6^	0.1	Antitumour efficacy of irradiations was not affected by use of hypofractionated regimens; FLASH and CONV irradiations were equipotent in the delay of GBM tumour growth in mice. The discrimination index of mice exposed to FLASH irradiations in the 10 Gy, 2 × 7 Gy, and 3 × 10 Gy groups showed no significant difference to unirradiated controls.

* NM = Not mentioned, ** ELBE = electron linac for beams with high brilliance and low emittance, *** DSB = double-strand breaks **** GBM = glioblastoma.

### In vivo studies of tumour treatment

Favaudon et al. (2014) demonstrated a sparing effect in mice after bilateral thorax exposure to single pulse, FLASH dose rate electrons (60 Gy/s, 4.5 MeV). Mice exposed to a total dose of 17 Gy at 0.03 Gy/s, representative of a conventional clinical dose rate, exhibited fibrogenesis initiation at 8 weeks post-irradiation, and progressing to intraparenchymal fibrosis at 34 weeks. Conversely, exposure to 60 Gy/s electron-FLASH (same total dose) did not develop pulmonary fibrosis after treatment. Activation of the Transforming Growth Factor Beta (TGFβ) cascade, a characteristic pathway in fibrosis pathogenesis, was also prevented. In subsequent dose escalation experiments, 30 Gy absorbed electron dose was the minimum required dose to induce fibrosis at FLASH dose rates. The relative biological effectiveness of both dose rates was comparable; growth of HBCx-12 A and HEp2 tumour xenografts was inhibited irrespective of FLASH or CONV dose rates, although FLASH irradiated mice exhibited a skin sparing effect [18].

### Zebrafish studies

Montay Gruel et al. (2019) displayed a FLASH sparing effect in zebrafish embryos [36]. In this study, alterations to the length of FLASH irradiated zebrafish embryos was significantly less than CONV irradiated embryos 5 days postfertilization, where irradiations occurred at 4 h postfertilization. When zebrafish were preincubated with an antioxidant, FLASH exhibited no further sparing effect, whilst the embryos were spared from CONV induced radiation damage [36]. Another zebrafish embryo model was used by Pawelke et al. (2021) to validate whether the pulse dose rate and oxygen levels used during irradiations masked a potential FLASH effect. 26 Gy of 20 MeV electrons at a continuous, conventional dose rate of 0.1116 Gy/s were compared to FLASH with a mean dose rate of 1 × 10^5^ Gy/s, and zebrafish embryos were irradiated within vessels at differing pO_2_ levels. A sparing effect was observed in this study at pO_2_ levels below atmospheric oxygen levels (< 148 mmHg) with a greater degree of protection afforded at < 5 mmHg (hypoxic conditions). Whilst FLASH-RT appeared to have a mild protective effect over CONV-RT at both pO_2_ levels, at high pO_2_ levels, FLASH and CONV-RT zebrafish morphologies exhibited minimal differences, whereas the effect was more substantial at low pO_2_ [48]. This included preventing the reduction of embryo length and eye diameter by 4%, as well as a 20% reduced rate of PE and SC in comparison to low dose rate, quasi-continuous irradiation.

### Mice brain studies

With the prospect of FLASH minimising healthy tissue damage, its effectiveness in the treatment of brain tumours is of extreme relevance clinically, particularly in paediatrics. In this respect, in vivo whole brain irradiation electron-FLASH studies are prevalent. Alaghband et al. (2020) displayed an apparent radioprotective effect in brain tissue of mice following FLASH irradiation. After exposure to 8 Gy, 6 MeV electron irradiation at 4.4 × 10^6^ Gy/s (FLASH), neurocognitive test results were indistinguishable from the control group, whereas conventional irradiation at 0.077 Gy/s with 6 MeV electrons caused substantial cognitive detriment. The benefit of FLASH, when utilised in the brain, was reduced neurocognitive impairment, attributed to the preservation of neurogenetic niche and neurogenesis. Mice irradiated at conventional dose rates observed a lower proportion of mature and immature neurons 4 months after irradiation. Additionally, a two-fold reduction in plasma growth hormone expression was also observed a week post treatment in comparison to controls, suggesting conventional irradiation also impairs pituitary gland function. These side effects were not observed in FLASH-irradiated mice [38]. Other whole brain irradiation studies displayed similar findings at and exceeding 100 Gy/s [32], including but not limited to: 200 Gy/s, 300 Gy/s [37], 2.5 × 10^3^ and 5.6 × 10^6^ Gy/s [39, 41].

### Higher order animal models

Demonstration of the electron FLASH effect is not limited to in vivo mice experiments, with implications in both mini-pig test animals and cat patients. Mini-pig irradiation at 300 Gy/s compared to 0.083 Gy/s markedly reduced damage to skin tissues, avoiding signs of acute toxicity including inflammation, ulcer formation and hair follicle destruction [33]. Additionally, late skin fibronecrosis was limited to CONV irradiated pigs, with FLASH experiencing no tissue complications at 36 weeks after 28–34 Gy irradiations. Six cat patients diagnosed with squamous cell carcinoma of the nasal planum were also FLASH treated, resulting in a progression free survival (PFS) of 84% at 16 months [33]. Side effects were limited to acute mucositis in half of the patients, and depilation across all cat patients. Although the sample size of this study is limited, it is some of the first evidence displaying no notable toxicity of FLASH in higher mammal models. One key limitation is that there are no experimental groups within this study irradiated at conventional dose rates; only retrospective data are referred to in drawing conclusions in this aspect. PFS ranged from 50 to 80% within other conventional dose rate trials [49]. This is promising in its potential applications in human patients, with regards to increasing total dosages (thereby improving tumour control) whilst also mitigating side effects.

Recently, a similar in vivo study by Kondradsson et al. (2021) attempted to identify potential adverse effects and treatment procedures required to administer electron-FLASH in canines with microscopic residual disease and spontaneous superficial solid tumours. Ten patients were prescribed doses ranging from 15 to 35 Gy, with average dose rates ranging between 400 and 500 Gy/s. 11 out of 13 irradiated tumours presented with either a complete response, partial response, or stable disease. Out of the 10 patients, only one experienced a grade 3 adverse skin event after a 35 Gy dose to the nasal planum, characterised by moist desquamation. Other adverse events appeared to be mild during follow up examinations 3–6 months post-treatment, including alopecia, dry desquamation, and erythema [50]. Akin to the feline-based study conducted by Vozenin et al., the key limitation to this preclinical trial was no comparative conventional dose rate group to compare adverse effects and disease response efficacy. It would be greatly beneficial for future in vivo work with canine and feline models to include conventional dose rate groups so that more accurate comparisons may be drawn. However, the feasibility in completing comparative experiments such as these may be difficult due to ethics considerations.

### First-In-Human case study

**Fig. 2:**
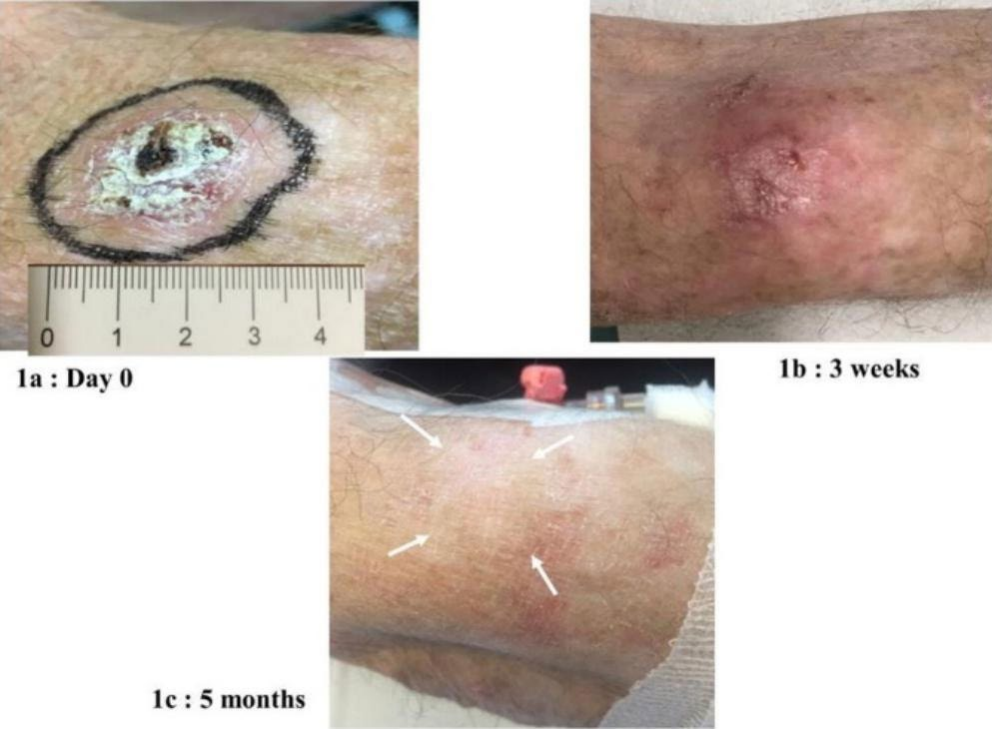
Temporal evolution of the treated lesion: (a) before treatment; the limits of the PTV are delineated in black; (b) at 3 weeks, at the peak of skin reactions (grade 1 epithelitis NCI-CTCAE v 5.0); (c) at 5 months. Reprinted from Bourhis et al. 2019 [34], Copyright (2019), with permission from Elsevier

The first-in-human case study of FLASH irradiation was reported in 2019 on a 75-year-old male with a multi-resistant cutaneous lymphoma, which had metastasised to multiple sites across his skin’s surface [34]. The patient had experienced unfavourable side effects from the previous treatment of approximately 110 tumour sites with either MV/kV X-rays or low energy electrons. Regardless of the relatively low radiation doses and fractionation regimens employed (20 Gy in 10 fractions or 21 Gy in 6 fractions), skin continued to respond poorly, requiring up to 4 months to heal damage at irradiation sites 3–4 cm in diameter. FLASH was therefore administered due to its previous implications in the sparing of healthy tissues whilst maintaining tumour control efficacy comparable to that of conventional dose rates. In this trial study, 5.6 MeV electrons at a dose rate of 167 Gy/s and dose of 15 Gy was administered to one of the patient’s most progressive ulcero-infiltrating tumours 3.5 cm in diameter. A 5 mm bolus was utilised, resulting in a 90% isodose coverage depth of 1.3 cm. Grade 1 epithelitis presented at 3 weeks post-FLASH, however 5 months after therapy almost all trace of negative skin reactions from treatment had receded (Figs. 2, [34]).

This study presents some valuable qualitative results depicting the temporal evolution of the treated area to provide comparisons between healthy and irradiated tissue. The fact that this is the first clinical study using FLASH dose rates is also substantial. However, it is limited in that they exclusively analysed the short-term effect of FLASH on cancerous skin tissue alone; the effect that electron FLASH would have on other organs of different tissue depths and morphologies based on this data is unknown. Regardless, this result bodes well for further clinical translation, as limiting inflammation is still greatly beneficial for patients experiencing radiation induced skin complications. Further translation clearly requires additional indications to display the benefits of this treatment modality, (e.g., irradiation of brain, lung, or liver tumours) would represent more complicated anatomy where improvement in outcomes is needed. Additionally, there were no conventional dose rate irradiations conducted within this study to directly compare the FLASH treatment to; all irradiations were FLASH only [34].

## Proton-FLASH

### In vitro studies

Although proton-FLASH studies are not as widespread as those conducted with electrons or X-ray photons, many of these models also reinforce the relationship between ultra-high dose rates and normal tissue protection. It is of note that save for proof-of-concept dosimetry planning by Wei et al. [51] and 6 experimental studies [52][53–57], herein to our knowledge, that no other papers either explicitly state or utilise the high LET Bragg peak region for proton-FLASH irradiations.

Buonanno et al. (2019) presented one of the first studies to analyse the long-term radiobiological effects of FLASH proton irradiation in vitro, particularly in non-cancerous human cells. At three different instantaneous dose rates: 0.05 Gy/s, 100 Gy/s or 1000 Gy/s, IMR90 lung fibroblast cells were irradiated with 0–10 Gy of 4.5 MeV protons and a colony forming assay conducted. Altering dose rate did not appear to have a dramatic impact upon cell survival, with no significant difference between the fraction of surviving cells across all dose rates and total doses administered. This aligns with previous studies observing the effect of proton dose rate on cellular survival, with dose rates exceeding 10^9^ Gy/s having no effect on colony formation compared to conventional dose rates for Human umbilical vein endothelial cells (HUVEC) [58], V-79 Chinese Hamster cells [59], or human-hamster hybrid cells (A_L_) [60]. Interestingly, this effect carries over into cancer cell clonogenicity, with human derived cervical cancer cell line HeLa [61, 62] exhibiting no dose rate dependence upon cellular survival post irradiation.

In quantifying DNA damage, exposure to 20 Gy absorbed proton dose delivered at a FLASH instantaneous dose rate of 1000 Gy/s displayed a statistically significant reduction in γH2AX fluorescence 30-minutes post-irradiation compared to both 0.05 Gy/s and 100 Gy/s protons [63]. Although this finding initially appears promising, it is limited by the immunolabelling techniques used to acquire data. Specifically, the resolution at which microscopy was performed (40x) and an apparent plateau of γH2AX fluorescence for doses ≥ 10 Gy suggests that accurate distinction of foci could not be obtained due to saturation above this threshold. At a dose of 20 Gy, approximately 700 double-strand breaks (DSBs) could be expected considering the formation roughly 35 DSBs/1 Gy [64]. One may assume that such a high number of γH2AX foci would prevent definitive resolution of the true number of DSBs formed, and thus lead to the saturation observed ≥ 10 Gy. This is a factor that needs to be taken into serious consideration in future work, as this will determine the doses at which the FLASH effect maximally prevents normal tissue DNA damage. Additionally, these results seem to contradict other studies analysing dose rate effects on DSB production via γH2AX [65–67] and 53BP1 [66, 68] immunofluorescence, which show a non-significant induction of these foci between FLASH and CONV dose rates.

Further tests to quantify markers of cellular senescence and inflammation (at 20 Gy, 1000 Gy/s) also showed reduced proportions of β-galactosidase positive cells 1-month post-irradiation (40% fewer vs. CONV) and TGFβ1 induction 24 h and 1-month (4.4-fold and 4.3-fold decrease respectively) post-irradiation [63]. At 100 Gy/s, the only significant difference was ~ 20% fewer β-gal positive cells compared to CONV, which may suggest that higher dose rates more effectively mitigate long-term cellular senescence.

These data scratch the surface of which factors modulate the proton-FLASH effect, illustrating that tissue protection may be more heavily reliant upon modifying gene expression in response to radiation exposure, especially concerning inflammatory and senescent cellular pathways. A summary table outlining all systematically identified proton and carbon-FLASH publications is presented in Table 3.

**Table 3:** Summary table of systematically identified particle-FLASH literature. For FLASH irradiations, delivery time, pulse or bunch count and rate for synchrotrons and cyclotrons respectively (or either for linear accelerators, (LINAC) or laser-accelerated protons (LAP)), average dose rate per fraction, instantaneous dose rate (within one pulse or bunch), and comparative CONV dose rate are provided. Data listed in bold have been calculated from information provided in the text. Only in vitro and in vivo works are listed. *In silico* and preclinical studies are summarised throughout the Proton-FLASH, Carbon-FLASH and Discussion sections

Ref.	Year	Particle Type, Source, Delivery Mode	Model	Energy (MeV)	Dose (Gy)	Delivery Time (s)	Pulse/Bunch Count	Pulse/Bunch Rate (Hz)	FLASH Dose Rate Average (Gy/s)	FLASH Instantaneous Dose Rate (Gy/s)	Low or CONV (Gy/s)	Outcome
[62]	2009	Proton, LAP pulsed and continuous	Human derived cervical cancer cell line HeLa	20	3	1 × 10^− 9^	1	3.9 × 10^4^	**3 × 10** ^**9**^	**3 × 10** ^**9**^	**30**	Pulsed FLASH (3 × 10^8^ Gy/s) RBE** was not significantly different than continuous (30 Gy/s) CONV irradiation. Additionally, there were also no substantial differences in the production of micronuclei observed between FLASH or CONV dose rates.
[61]	2011	Proton LAP pulsed and continuous	Human derived cervical cancer cell line (HeLa)	20	0–5	1 × 10^− 9^	1	100	**1 × 10** ^**9**^	**1 × 10** ^**9**^	**30**	Reduced number of cells accumulated in the G_2_ phase of the cell cycle 10 h post-FLASH compared to post-CONV. No differences in HeLa cell clonogenicity were observed when comparing FLASH and CONV dose rates.
[60]	2011	Proton, LAP, pulsed and continuous	Human-hamster hybrid cells (A_L_)	20, 23, 25	3.6	1 × 10^− 9^	1	3.9 × 10^4^	**3.6 × 10** ^**9**^	**3.6 × 10** ^**9**^	**36**	RBE** showed no significance between pulsed (FLASH) and continuous (CONV) modes. Higher proportion of dicentric, centric rings and excess acentrics chromosome aberrations in CONV mode compared to FLASH.
[59]	2012	Proton, LAP, pulsed and continuous	Chinese hamster cells (V-79)	1–5	0.8–5	7 × 10^− 13^	1	NM*	1 × 10^9^	1 × 10^9^	NM*	Laser accelerated protons at FLASH dose rates appear to show no substantial difference in RBE (1.4 ± 0.2) in terms of clonogenicity when compared to previous studies utilising protons at CONV dose rates.
[67]	2012	Proton, LAP, pulsed and continuous	Human derived cervical cancer cell line HeLa	20	1, 5	1 × 10^− 9^	1	3.9 × 10^4^	**1 × 10**^**9**^, **5 × 10**^**9**^	**1 × 10**^**9**^, **5 × 10**^**9**^	**10, 50**	No significant difference observed between FLASH (pulsed) and CONV (continuous) proton irradiation of HeLa cells regarding both the formation and time-dependent loss of γH2AX foci.
[54]	2014	Proton LAP, pulsed and continuous	NMRI (nu/nu) mice model, FaDu tumour xenograft	23	20	2100	1	3.9 × 10^4^	**0.009**	**2 × 10** ^**10**^	**0.002**	In the treatment of human tumour xenografts, FLASH and CONV dose rate protons appeared to have comparable measures of both tumour growth delay and RBE (1.22 ± 0.19 and 1.10 ± 0.18 respectively). Note that although instantaneous dose rates differ greatly, average dose rates are similar due to the 35 min required to irradiate tumour volumes.
[66]	2016	Proton, LAP: pulsed LINAC: continuous	Human lung cancer cell line A549	0–2.2	0, 0.25, 0.5, 1, 2	NM*	NM*	NM*	NM*	1 × 10^8^	0.01	Laser and conventionally accelerated protons induce γH2AX and 53BP1 foci with similar efficacy. Induction of 3-nitrotyrosine, a marker for nitroxidative stress, was 2-3-fold lower in laser accelerated protons compared to conventionally accelerated protons, with similar induction compared to X-rays at 1 Gy.
[58]	2017	Proton, LAP: pulsed and continuous	Human umbilical vein endothelial (HUVEC) cells	6–14	0–4.5	**1.5 × 10** ^**− 9**^ **– 4.5 × 10** ^**− 9**^	NM*	NM*	**> 1 × 10** ^**9**^	**> 1 × 10** ^**9**^	NM*	No statistically significant difference in HUVEC clonogenic survival between laser-accelerated protons, conventionally accelerated protons and X-rays. Conversely, laser-accelerated protons saw higher proportions of senescent cells at 7 and 14 days after 0.5 Gy, and from 2–28 days after 1.5 Gy irradiations. No significant effect on senescence for 4.5 Gy.
[68]	2019	Proton, LAP: pulsed, cyclotron: continuous	Human skin fibroblasts (AG01522B)	10	1–2	1 × 10^− 9^	1	NM*	1 × 10^9^	1 × 10^9^	**0.067**	Non-significant differences between 53BP1 foci induction, foci size, and fraction of repaired DSBs between FLASH laser-accelerated protons and 225 kVp X-rays. However, protons appeared to have greater yields of 10–14 foci per cell at 6 and 24 h post irradiation. Track structures (foci/cell/track) of laser-accelerated protons were also similar to cyclotron-accelerated protons.
[63]	2019	Proton, LINAC: pulsed	Normal human lung fibroblasts (IMR90)	4.5	0–20	NM*	NM*	NM*	*NM	100, 1000	0.05	For colony formation assay, Clonogenic cellular survival was not affected by proton dose rate regardless of dose administered. Decrease in γH2AX foci at 20 Gy for 1000 Gy/s, 100 Gy/s FLASH foci formation comparable to 0.05 Gy/s CONV. Cell senescence and expression of TGFβ1 decreased with increasing dose rate (statistically significant).
[65]	2019	Proton, LAP: pulsed, cyclotron: continuous	Glioblastoma cell lines SF763 and U87-MG, human colon carcinoma (HCT116)	20	0–10	NM*	0–12	0.5	NM*	1.5 × 10^8^	**0.021**	Laser driven protons (LDP) induce numbers of γH2AX foci similarly to conventional, continuous proton beams and X-rays. Modification of the LDP repetition rate altered the survival of both WT and p53^−/−^ HCT116 cells. Survival was a non-monotonic function of time between pulses. Inhibition of PARP1 protein reversed this effect.
[69]	2019	Proton, cyclotron: quasi-continuous	Zebrafish embryos	224	0–45	**0.1–0.45**	NM*	NM*	100	5 × 10^4^	0.08	Statistically significant difference between FLASH and CONV protons at 25 Gy, with a lower incidence of pericardial edema for FLASH irradiated embryos. Dose rate did not influence relative survival or spinal curvature as a function of total dose.
[70]	2019	Proton, NM*	C57BL/6J mice model, Lewis lung carcinoma	NM*	18	NM*	NM*	NM*	40	NM*	NM*	Recruitment of CD3+, CD4 + and CD8 + T-lymphocytes from the tumour periphery to inside the tumour volume was increased under FLASH irradiation. Tumours were significantly smaller in the FLASH or pulsed-FLASH groups compared to CONV-RT.
[71]	2019	Proton	C57BL/6 mice model, thorax irradiation	NM*	15, 17.5, 20	0.375–0.5	NM*	NM*	40	NM*	0.5	Female mice irradiated with FLASH exhibited improved overall survival, breathing ability, and reduced incidence of dermatitis compared to CONV irradiations at doses of 17.5 and 20 Gy. There was minimal difference between FLASH and CONV in male mice. Mode of cell death was also affected between a pulse-FLASH mode, FLASH and CONV irradiations, regardless of mice gender.
[72]	2020	Proton, cyclotron, quasi-continuous	C57BL/6J mice, abdominal irradiation, MH641905 mouse pancreatic tumour cells	230	12, 15, 18	**0.16–0.29**	18–20	1.06 × 10^8^	63, 94	**~ 300**	0.71, 0.74, 0.94	Whole abdominal 15 Gy FLASH irradiations substantially prevented the loss of proliferating cells (higher proportion of EdU + cells) within intestinal crypts compared to CONV irradiation. Conversely, 12 and 18 Gy FLASH irradiation of tumours did not exhibit a tissue sparing effect.
[73]	2020	Proton, cyclotron: quasi-continuous	C57BL/6J mice model, partial abdominal irradiation	225.5	13, 16, 19, 22	**0.096–0.181**	NM*	1.06 × 10^8^	106–137.88	NM*	< 0.745	Mice exposed to 16 Gy FLASH exhibited higher overall survival 21 days post-irradiation (100%) compared to CONV (40%). At 19 and 22 Gy doses regardless of dose rate, all mice died by 12 days post irradiation. FLASH mice also showed improved weight retention following irradiation compared to CONV. Chronic inflammatory cell infiltration and thickening of submucosa and muscularis was observed at 16 Gy under both FLASH or CONV conditions.
[74]	2021	Proton, cyclotron: continuous	C57BL/6J mice model, toxicity model: hind leg irradiation, tumour model: murine derived squamous cell carcinoma (MOC1/MOC2)	250	15, 35	0.24–0.61	NM*	7.2 × 10^7^	61.8, 62.2, 115.1	206.3, 207.6, 385	1	In immunocompetent mice, equivalent tumour control observed between FLASH and CONV-RT. Decreased TGFβ1 production in FLASH irradiated mice 24- and 96-hours post-irradiation. G-CSF, and GM-CSF cytokine ratio higher in FLASH mice than CONV, indicative of reduced toxicity.
[75]	2021	Proton, cyclotron: continuous	C57BL/6 and C3H/HeJ mice model: *LSL-Kras*^*G12D/w*t^;*p53*^*FL/FL*^ derived fibrosarcoma and RIF mice sarcoma. Canine model, skin irradiation	230	30, 45	**0.24–0.65**	NM*	NM*	69–124	NM*	0.39–0.65	Mice exposed to 30 Gy FLASH irradiation had higher overall survival and protection from severe morbidities (mean survival > 249 days) compared to CONV irradiation (mean survival = 211 days). RNA-seq of FLASH irradiated murine skin displayed increased expression of genes responsible for vascular repair, contrasting CONV which displayed upregulation of apoptotic and keratin signalling. In skin irradiation studies of both mice and canine models, FLASH alleviated skin damage and inflammation and reduced TGFβ1 expression compared to CONV. There was no dose rate dependence on tumour kill efficacy in the treatment of mice sarcomas.
[52]	2021	Proton, cyclotron: continuous	C57BL/6J mice, whole abdominal irradiation, MH641905 mouse pancreatic tumour cells	230	15 ± 0.1, 18 ± 0.1	**0.138–0.167**	NM*	NM*	108 ± 12.3	NM*	0.82 ± 0.196	15 Gy FLASH abdominal irradiations resulted in a higher percentage of EdU+ (marker of cell proliferation) cells per crypt and regenerated crypts compared to CONV. No statistically significant difference between utilisation of the SOPB*** or entry ‘plateau’ region of proton beam upon EdU + cells or crypt regeneration at FLASH or CONV dose rates. 18 Gy tumour irradiations resulted in similar tumour control between dose rates, however radiation lethality 20 days post irradiation was substantially decreased in FLASH SOBP*** treated mice (15%) vs. CONV (70%).
[53]	2021	Proton, synchrocyclotron, pulsed	C57BL/6 mice, total abdominal irradiation	230	10–19	**0.1–0.2**	**80–152**	756	100	6200	0.1	Mice exposed to FLASH irradiation had a higher LD50 compared to those exposed to CONV at equivalent dosages. Survival was increased under FLASH conditions by between 10–20% depending upon dose administered.
[76]	2022	Carbon, synchrotron: pulsed	Chinese hamster ovary cells (CHO-K1)	280 ****	7.5	0.13	1	1.45 × 10^10^	70	70	0.6	A statistically significant increase in cell survival was displayed for CHO-K1 cells exposed to FLASH vs. CONV irradiation at 0.5% and 4% oxygenation. No significant difference was observed between dose rates for cells exposed to 21% oxygenation.
[77]	2022	Proton, cyclotron: continuous	CDF1 mice, hind limb irradiation	244–250	23.2–39.2	0.35–0.73	NM*	NM*	65–92	NM*	0.35–0.40	After irradiation of hind limb skin, FLASH dose rates exhibited a normal tissue sparing effect in comparison to CONV, with a 44–58% higher dose required under FLASH to produce comparable tissue toxicities to CONV irradiation. This is consistent amongst all levels of acute skin damage (1.5–3.5), with the dose necessary to produce moist desquamation in 50% of mice (MDD_50_) being higher in FLASH irradiated mice.
[55]	2022	Carbon, synchrotron: pulsed	Human lung fibroblasts (HFL1) and human salivary gland tumour line (HSGc-C5)	290 ****	1, 2, 3	**0.005–0.031**	1	NM*	96–195	96–195	8–13	FLASH and CONV irradiation of HFL1 cells exhibited no significant difference in post-irradiation impediment of cell growth and induction of senescence. This applied when using the entrance plateau region (13 keV/µm) and Bragg peak region (50 keV/µm) of the carbon beam. In HSGc-C5 cells, no significant difference was observed in clonogenic survival when comparing either FLASH or CONV dose rates, or when utilising the plateau or Bragg peak region of the beam.
[56]	2022	Proton, LINAC: continuous	Normal human lung fibroblasts (IMR90)	4.5	1.5, 15	**0.015–0.15**	1	NM*	100	100	0.33	Clonogenic survival of IMR90 cells was improved under FLASH conditions compared to CONV. Radiation induced oxidative stress 40 min post-irradiation was also substantially lower in FLASH irradiated cells compared to CONV. In mitochondria, FLASH samples displayed similar morphology to unirradiated controls, whereas CONV samples showed morphological abnormalities. Expression of mitochondrial fission protein Drp1 was found to be higher in CONV irradiated cells versus FLASH, where there was no significant difference in Drp1 expression compared to control samples. CONV-IR also induced a significant decrease in mitochondrial copy number in comparison to FLASH.
[57]	2022	Proton, synchrotron: pulsed	C57BL/6 mice, brain irradiation	146.6 ****	10	0.08	1	NM*	120	120	0.17	Under CONV, the fraction of γH2AX positive cells increased 10-fold compared to control, whereas under FLASH 2-fold increase was observed. In addition to significantly lower γH2AX radiation induced foci induction, FLASH conditions reduced the degree of inflammation associated with macrophage/microglia compared to CONV.
[78]	2022	Proton, cyclotron: continuous	Zebrafish embryos	224	32	0.115	1.2 × 10^7^	1.06 × 10^8^	300	1.5 × 10^3^	0.15	FLASH irradiations exhibited reduced rates of pericardial edema at day 3 (FLASH: 54.3 ± 18.9%, CONV: 67.6 ± 20.6%) and day 4 (FLASH: 75.3 ± 13.1%, CONV: 90.1 ± 9.1%) post irradiation. The same was observed for spinal curvature at day 3 (FLASH: 15.4 ± 10.3, CONV: 27.2 ± 12.0) and day 4 (FLASH: 24.6 ± 11.5, CONV: 38.2 ± 15.5).
[79]	2022	Carbon, synchrotron: pulsed	C3H/He mice, LM8 osteosarcoma model	240 *****	18	0.15 ± 0.02	1	NM*	100	100	0.3	Under either FLASH or CONV dose rates, tumour control was achieved with similar efficacy. However, under FLASH dose rates, the proportion of mice with lung metastases was significantly lower than those irradiated under CONV (FLASH: ~10%, CONV: ~30%, Control: ~40%). Irradiated muscle tissues exhibited disorganized morphology under CONV irradiation, which was greatly reduced in FLASH irradiated mice.
[80]	2022	Proton, cyclotron: continuous	CDF1 mice, CH3 mouse mammary sarcoma model	250	40–60	0.449–0.845	NM*	NM*	71–89	NM*	0.33–0.63	The total dose for 50% tumour control (TCD_50_) for either dose rate was comparable, FLASH: 51.3 Gy, CONV: 49.1 Gy. For mice with tumour control, radiation induced fibrosis was mitigated in FLASH irradiated mice, with moist desquamation presenting in 50% of mice (MDD_50_) at 52.3 Gy for FLASH versus < 40.1 Gy for CONV, and fibrosis presenting in 50% of mice (FD_50_) at 55.6 Gy for FLASH versus 48.6 Gy for CONV.
[81]	2022	Proton, cyclotron: continuous	Zebrafish embryos	235	9–27	**0.007–0.3**	NM*	7.29 × 10^7^	90, 1260	NM*	0.1, 0.9	In measuring radiolytic production of peroxide in a water phantom mimicking zebrafish physoxia (4% O_2_), the overall yield of H_2_O_2_ was reduced under FLASH conditions regardless of dose administered. There was no substantial difference in zebrafish viability or fish length between FLASH and CONV irradiation modes, and both irradiation modes sparing zebrafish morphology compared to photon and electron irradiation at a dose of 10 Gy.
[82]	2022	Proton, LAP: pulsed	Prostate tumour cells PC3, normal prostate epithelial cells RWPE1	2	7.4–37.1	50–150	10–30	0.2	0.15–0.25	2.3 × 10^7^ – 3.8 × 10^7^	NM*	The surviving fraction of normal prostate RWPE1 cells irradiated by FLASH dose rate protons was greater than one order of magnitude higher than that of tumour prostate PC3 cells at a dose of 7 Gy. Increasing dose above approximately 15 Gy resulted in a small reduction of RWPE1 survival, whilst PC3 cells did not survive at any doses above this value.

* NM = Not mentioned, ** RBE = Relative Biological Effectiveness, *** SOBP = Spread-Out Bragg Peak, **** Units = MeV/u, ***** Units = MeV/n.

### In vivo studies

One of the first papers to demonstrate in vivo FLASH radioprotection using protons was published in 2020 by Diffenderfer et al. [72]. A 230 MeV proton beamline with dose rates of 78 Gy/s for FLASH and 0.9 Gy/s for conventional irradiation were utilised in 15 Gy whole abdominal irradiations of C57BL/6J mice. Akin to studies performed with electron irradiation, FLASH irradiated mice exhibited greatly reduced levels of acute intestinal damage, defined by an increased number of EdU positive cells within abdominal crypts and increased crypt regeneration compared to the conventional dose rate. Moreover, where symptoms of fibrosis were severe in conventionally irradiated mice, FLASH irradiated tissues displayed intestinal morphology similar to that of non-irradiated tissues 8-weeks post irradiation. In a follow up experiment testing for tumour growth and control probability in a flank injection, murine pancreatic cancer model, no difference was observed between either dose rate used and doses of 12 and 18 Gy [72]. This aligns with previous claims that proton-FLASH has comparable tumour kill efficiency to conventional dose rates. Yet, this data is exemplary in highlighting the favourability of ultra-high dose rates to both mitigate the inflammatory response and maintain cellular proliferation after exposure. A murine model conducted by Cunningham et al. also supports this, wherein equivalent tumour control was maintained in a squamous cell carcinoma FLASH irradiation compared to CONV [74]. Additionally, when FLASH irradiating normal hind leg tissue, there were decreased levels of TGFβ1 24- and 96- hours post irradiation, as well as a higher G-CSF to GM-CSF ratio, both indicative of reduced inflammation compared to CONV [74].

Similar experiments were performed by Zhang et al. (2020) utilising an experimental beamline for whole abdominal irradiations of mice, with average dose rates of 120 Gy/s and 0.05 Gy/s for proton-FLASH and CONV respectively. Mice survived doses of 13 Gy at either dose rate and died within 15 days of 19 and 22 Gy irradiation. However, an apparent differential effect was observed for 16 Gy irradiations, with 100% of FLASH mice surviving versus ~ 40% at the CONV dose rate. In addition, FLASH mice exhibited better weight retention 9 days post-irradiation. Hematoxylin and eosin stains were also performed to observe late effects 90 days post-irradiation. Both dose rates showed mild signs of inflammation with inflammatory cells infiltrating intestinal villi. Remarkably, FLASH irradiated intestines appeared to exhibit reduced signs of inflammation compared to CONV protons, evidenced by a lower proportion of infiltrating inflammatory cells and thinner layer of hyperplastic submucosa and muscularis [73]. In syngeneic mice models, there is evidence that proton-FLASH produces an improved tumorigenic response due to increased recruitment and infiltration of CD3 + T cells within the tumour microenvironment [70]. These findings may be reflective of the ability of FLASH to minimise interference with, or perhaps aid in stimulating, the immune response.

Similar to the previously described electron-FLASH experiment conducted by Pawelke et al. [48], Beyreuther et al. [69] also performed zebrafish embryo irradiations, but with 224 MeV protons at a CONV dose rate of 0.08 Gy/s and FLASH at 100 Gy/s. The endpoints of this study were relative embryonic survival and the rate at which morphological changes occurred, including pericardial edema (PE) and spinal curvature (SC). Only 23 Gy irradiation appeared to exhibit statistically significant differences between CONV and FLASH embryos with PE at 3- and 4-days post-irradiation; dose rate did not appear to have a substantial effect upon any other endpoints or at other doses [69]. Akin to in vitro studies, the Bragg peak is not reported to conform to targeted tissues throughout these experiments.

### First-In-Human clinical trial

In December of 2020, recruitment began for the first proton-FLASH clinical investigation, conducted at Cincinnati Children’s Proton Therapy Center [83]. The focus of this study was to assess the workflow of a palliative FLASH treatment for bone metastases in the extremities of 10 patients aged 18 years or older. FLASH treatments were delivered at a dose rate of 51–61 Gy/s, with single dose regimen of 8 Gy being utilised on a total of 12 metastatic sites across all patients. This was followed by assessment of radiation related normal tissue toxicities and adverse side effects in addition to pain response, use of pain relief and pain flare [84]. Key results from the study include 6 out of the 12 treated sites experiencing a complete pain relief response, 2 of 12 sites reporting partial pain relief, and 4 of 12 sites experiencing pain flare following treatment. Twelve adverse events were reported within this patient group, with eleven of these being classed as grade 1 including skin hyperpigmentation, edema, erythema, fatigue, and puritus. One patient also experienced grade 2 extremity pain 1 month post treatment. FLASH was deemed clinically feasible in the palliative treatment of bone metastases, with the efficacy of treatment and the profile of adverse effects being analogous to conventional dose rate radiotherapies regimes [85].

## Carbon-FLASH

The representation of experimental carbon-FLASH literature, in a radiobiological context, is sparse, with only six papers fitting the systematic criteria of this review. Two of these papers, authored by Zakaria et al. [12, 86], outline the potential anti-tumour benefits of carbon FLASH *in silico*. Monte Carlo simulations produced 3-dimensional track segments of low and high LET carbon ions to draw comparisons between their energy deposition profiles. High LET, 4.1 MeV/nucleon carbon ions (~ 330 keV/µm) exhibited a significant production of radiolytic oxygen after movement of the ions through water compared to lower LET carbon ions. As molecular oxygen can act as a radiosensitiser, they suggest that this radiolytic formation of oxygen at high LET (i.e., Bragg peak) may offer enhanced tumour control. Oxygenation remains relatively unchanged within the low LET, ‘normal tissue’ region in these simulations, also suggesting that the sparing capacity of carbon-FLASH would be unchanged [12]. In the first in vitro study by Tinganelli et al., CHO-K1 cells exposed to 7.5 Gy of 70 Gy/s carbon-ions had greater surviving fractions at 0.5% and 4% oxygenation (hypoxia) post-FLASH compared to CONV, but not at 21% O_2_ (physoxia). These irradiations were performed within the plateau region of the dose-depth distribution (~ 13 keV/µm), with minimal sparing even at lower oxygen concentrations [76]. Following this, Tinganelli et al. published the first in vivo carbon FLASH study, analysing normal and tumour response in a C3H/He mouse model. Notable findings include comparable tumour control under either the FLASH (100 Gy/s) or CONV (0.3 Gy/s) dose rates, however there was a substantial reduction in the proportion of mice with lung metastases (~ 10% of mice under FLASH versus ~ 30% under CONV). In normal tissues, morphology was greatly spared under FLASH conditions compared to CONV, providing further evidence in support of a FLASH effect when applying carbon ions at ultra-high dose rates in vivo [79].

Additional simulation work explores the interaction of multiple interacting carbon ion tracks, instantaneous irradiations, and the effect these have upon radiolytic oxygen consumption and peroxide ion formation. At the highest dose rate utilised of approximately 10^10^ Gy/s, 300 MeV/nucleon (~ 11.6 keV/µm) carbon ions consumed 90% of oxygen present in solution, suggesting that carbon ions at FLASH dose rates are capable of inducing transient intracellular hypoxia [86]. Intriguingly, they also show that peroxide ion formation increases with increasing dose rate, and draw comparison to previous work conducted by Montay-Gruel et al. [36] where lower concentrations of H_2_O_2_ were produced at ~ 1000 Gy/s compared to ~ 0.1 Gy/s. However, as the dose rates and particles utilised (carbon ions vs. electrons) between these studies differ so greatly, it is very difficult for accurate comparisons to be drawn.

## Mechanisms of the FLASH effect

Data from studies reviewed thus far collectively indicate three potential mechanisms for enhancing tumour control probability against the normal tissue complication probability during FLASH irradiation with particles. Specifically, normal tissue sparing effects due to rapid oxygen depletion; increased complexity in the nature of DNA damage that is more easily repaired by normal cells; and induction of antitumour immune response. Thus, the FLASH effect may both reduce normal tissue toxicity as well as enhance tumour kill efficiency. While these mechanisms are the most frequently proposed and investigated [13, 87], data are not outright conclusive and, as is typical for radiobiology, likely to be highly convoluted and complex. Research captured by the systematic review specifically contributing to the discussion of these mechanisms are reviewed in the following sub-sections.

### Oxygen depletion and reactive oxygen species

The cell killing capability of radiation therapy relies upon causing irreversible DNA damage to cancerous cells. When any cell is exposed to ionizing radiation (including protons) energy is transferred to intracellular chemical species upon physical interaction along its track. One of the sources of damage for particles is via direct collision and consequent ionization of DNA. Indirect DNA damage can also occur via the interaction of ionizing radiation with the intracellular species surrounding it, including oxygen and water [88]. Energetic charged particles can dissociate water and other molecules to form reactive oxygen species (ROS), DNA-damaging molecules which can disrupt DNA nucleotide sequences or the sugar-phosphate backbone [89]. It is suggested that the production of ROS is limited during FLASH irradiation, the hypothesis being that local oxygen is rapidly depleted at ultra-high dose rates, quicker than reoxygenation can occur. This means that any extra dose within the short timeframe does not contribute to further ROS production, thereby avoiding further damage to DNA due to this mechanism. With respect to normal tissues this leads to a state of radio-resistance. Within a hypoxic tumour environment however, then the change in radiosensitivity is less pronounced.

**Fig. 3:**
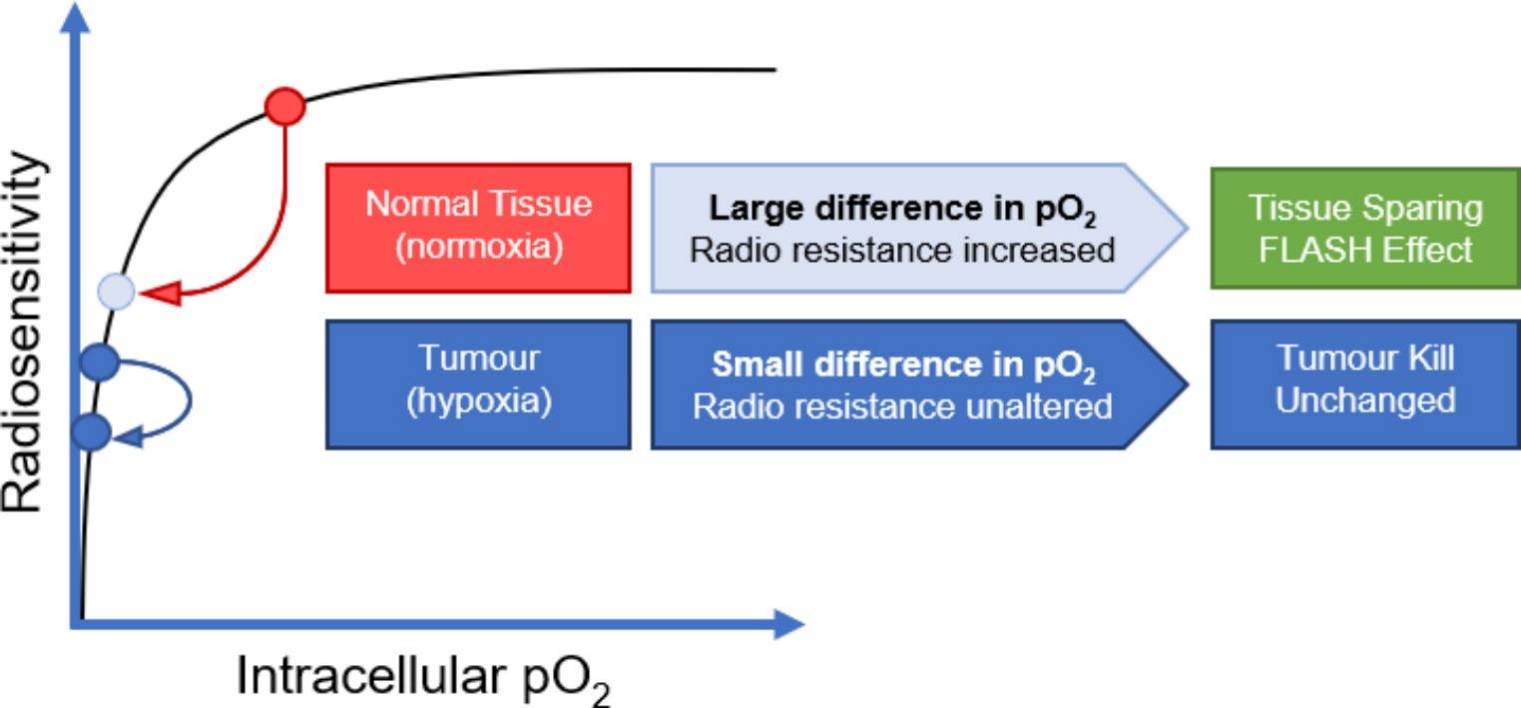
Representation of the oxygen depletion hypothesis, depicting relative radiosensitivity of normal versus tumour tissues as a function of oxygen tension. Normal tissues see a more significant change in intracellular O_2_ levels, affording a brief period of hypoxia which protects normal tissues from ROS related damage during FLASH irradiation. On average, cancerous cells are already relatively hypoxic, so radiation resistance is unchanged by O_2_ consumption via FLASH. Permitted reprinting from Wilson et al. 2020 [87]

Solid tumours are often deficient in cellular oxygen due to poor vascularisation, meaning fewer ROS can be produced. As a result, hypoxic tumours are 2–3 times more radioresistant than under normoxic conditions [90]. By the oxygen-depletion theory, then the difference in intracellular O_2_ is not as substantial for tumorigenic tissues (hypoxic à hypoxic) compared to normal tissues (physoxic à hypoxic) after FLASH exposure, meaning this protective effect is exacerbated in healthy tissue compared to cancerous tissue (Fig. 3).

Although this theory holds credence for in vitro models of oxygen depletion, there is debate in the radiobiology community as to the translation of this phenomena in vivo [91]. If a region of cancerous tissue has comparable levels of oxygenation to normal tissues, then would one not expect a tumour sparing effect as well? The controversy surrounding this theory suggests that transient oxygen depletion during FLASH is not the prime mechanism responsible for healthy tissue sparing, and that there are multiple other factors that contribute towards this radiobiological outcome [12, 92].

### DNA damage: repair and response

The frequency of DSBs present within irradiated cells is one of the primary indicators of radiation induced DNA damage. Understanding the differences between how normal and tumour cells accumulate and respond to DSBs is therefore pivotal in defining the radiobiological characteristics of FLASH. Reports have indicated that ultra-high dose rates may reduce the number of γH2AX foci in both normal human fibroblasts and mice crypt base columnar cells, after administration of 20 Gy of 1000 Gy/s 4.5 MeV protons [63] or 14 Gy of 216 Gy/s 16 MeV electrons [43] respectively. This effect has also been illustrated in mouse Lewis lung carcinoma, observing a significantly higher number of γH2AX foci after 0.06 Gy/s versus 352 Gy/s 16 MeV electron irradiation [93]. However, clonogenic assays presented non-significant differences in cellular survival after 3 Gy irradiation. This appears contradictory to the equipotent antitumour effect ultra-high dose rates have in comparison to low dose rates; with present data, the implications of these findings are unclear.

Analysis and interpretation of γH2AX foci are fraught with challenges. One potential explanation for the differences between foci measurements and clonogenic survival is that although particle-FLASH reduces the total number of DSB foci observed in irradiated cells, the complexity of these DNA lesions increases with increasing dose rate [13, 94]. The pattern of DNA damage that FLASH irradiation produces compared to CONV has not yet been thoroughly analysed, and as such, there is difficulty in making conclusions concerning the degree of DNA damage that FLASH causes or prevents based upon clonogenic and γH2AX immunofluorescence assays alone. If under conventional irradiation conditions, DNA lesions are more broadly dispersed amongst foci, this would allow for resolving additional DNA DSBs during microscopic examination. Conversely, FLASH irradiation may produce clustered DSBs, where breakages occur within proximity to one another and are not discretely resolved. The initial conclusion for these observations would be that DNA damage appears to be of higher severity for CONV. Furthermore, γH2AX foci are indicators of the initiation of the DSB repair process, not of DSBs explicitly. If the nature of DNA damage between low and ultra-high dose rates differs, then these foci measurements are not directly comparable. This raises questions as to whether γH2AX foci assays can be presented as reliable predictors of cell death in this context.

Clustered DSBs are significantly more difficult for cells to repair than isolated lesions [95], and cancerous cells often contain deleterious mutations within DNA damage repair (DDR) pathways. Particularly, defects in the non-homologous end joining (NHEJ) and homologous recombination (HR) repair pathways, responsible for DSB repair, result in increased radiosensitivity [96], an effect which is exacerbated for proton irradiation [97]. Initial observations show that the key radiobiological difference in response to particle-FLASH is that normal cells can better withstand ultra-high dose rates compared to cancerous cells. It is possible that tumour kill efficacy is maintained regardless of a reduction in γH2AX foci formation due to the inherent differences in DDR factor expression between normal and cancerous tissues. Normal cells may have a favourable predisposition for processing the pattern of DNA damage FLASH induces due to being more genetically stable, maintaining the NHEJ and HR repair mechanisms responsible for overcoming otherwise lethal DNA damage [13, 98]. Due to the inherent genomic instability of cancerous cells, dysfunction of these mechanisms would prevent cell survival after exposure, as clustered DSBs would be accumulated almost instantaneously at multiple foci under particle-FLASH conditions. Therefore, the differential effect within normal and tumorigenic tissues may lie within their respective abilities to resolve DNA damage of increasing complexity. Characterising the activation of the DNA repair factors expressed in each cell type post-FLASH would aid in determining which pathways drive FLASH’s normal tissue sparing capabilities and provide explanations for FLASH’s unchanged tumour kill efficacy.

### Immune modulation: oncolytic and anti-inflammatory

Pathways involved in immune response are also thought to play a major role in both the enhancement of tumour control and reduction of inflammation during FLASH. It has been previously shown that ionizing radiation stimulates anti-tumour responses via expression of damage-associated molecular pattern (DAMP) molecules, consequently recruiting dendritic and cytotoxic T cells to neutralise tumour bodies [99, 100]. Indeed, the abscopal effect would be a prime example of radiation induced immune response, hypothesised to act via anti-tumour specific T-cells [101]. Yet, not much is understood concerning the implications of how FLASH interferes with the immune response in normal cells. Computational models predict that ultra-high dose rates may spare circulating immune cells, showing a reduction in immune cell death from 90 to 100% at conventional dose rates to 5–10% for dose rates greater than 40 Gy/s [102]. There is also evidence that TGFβ1, a pro-inflammatory cytokine, may be modulated during FLASH. After administration of 20 Gy at 1000 Gy/s (FLASH) or 0.2 Gy/s (CONV) protons to in vitro lung fibroblasts, Buonanno et al. observed a 4.7-fold reduced TGFβ1 induction during FLASH compared to CONV, suggesting that higher dose rates may be able to substantially reduce the degree of chronic inflammation in normal tissues [63]. Other factors, including modulation of the tumour microenvironment [70, 93], differential cytokine expression [74], and sparing of stem cell niches [44, 103] are also thought to contribute towards the FLASH effect, with further preclinical studies required to substantiate these observations.

## Discussion

Whilst current literature appears to be explicit in biological methodology and outcomes of particle-FLASH experiments, the reporting of dosimetry and beam parameters are often not as thorough. To draw more accurate comparisons and conclusions between different FLASH studies, there is a necessity for both more stringent quality control and detailed descriptions of the irradiation characteristics used in these experiments. Particle- and electron-FLASH papers cite usage of substantially high dose rates, with average and/or instantaneous dose rates on the order of 1 × 10^6^ Gy/s or higher [38, 41, 59–61, 65, 66, 68, 104]. This is potentially a considerable source of error in delivered dose as delivery of a conventional clinical dose (e.g., 50 Gy) in a matter of microseconds could have a significant degree of variability if FLASH pulses are not time gated with extreme precision, this being exacerbated at lower doses used experimentally. As this often appears to be neglected, there may be data which incorrectly correlates radiobiological effects to a predicted ‘dose’ for which there is substantial degrees of error. More thorough documentation of the methodology utilised to measure dosimetry needs to be provided, as it is intrinsically linked to the radiobiological data presented.

In considering the literature on potential therapeutic benefit of particle-FLASH, the data reviewed here illustrate differences in radiobiological responses compared to conventional irradiation dose rates. Although the effectiveness of particle-FLASH to eradicate tumours in vivo appears to be comparable to current clinical strategies, the outstanding result to date appears to be that the differential effect between normal and healthy tissues is increased considerably. Diffenderfer et al. [72] address these points effectively in their study, providing good justifications from past proton and electron-FLASH literature to form the basis of their experiments. One of the main limitations is that whilst protons are used for tumour irradiations instead of photons, their energy deposition profile is not taken advantage of, i.e. protons are transmitted through target volumes and rather than stopping as would be conducted clinically. Of these 34 particle-FLASH papers, only 6 utilise the Bragg peak for irradiations, the remaining employing the initial plateau region. Modulation of the Bragg peak and analysis of the damage that might be spared if FLASH dosage was conformed to the tumour volume is lacking in the proton studies mentioned, which is not reflective of clinical treatments using particle therapy that do employ this. For this technique to translate accurately from the preclinical phase to clinical use, it is vitally important that experiments mimic current clinical treatment methods as closely as possible. Ideally, this would include performing 3-dimensional dosimetry and conformation of the proton beam to a xenografted, orthotopic or spontaneous tumour volume. Nevertheless, this is not always feasible due to the limitations of the equipment used for irradiation or the scope of the experiments being performed (in vivo mice models for instance). This does not negate the value of transmission particle-FLASH experiments such as these, as they are invaluable in identifying how particle-FLASH affects healthy tissue proliferation and survival, as well as providing a general overview of which biological pathways respond to irradiation and in what manner. However, it should still be kept in mind that LET of the protons used in these studies and clinical practice differ substantially, and as such conclusions drawn from preclinical data should be taken with caution when attempting to translate them into clinical trials.

As mentioned previously, there is debate concerning which mechanisms contribute towards the particle-FLASH effect. Specifically, clinical scientists and some academics are uncertain whether the oxygen depletion hypothesis is valid, and if its contribution to normal tissue sparing in vivo is overstated compared to other hypothesised mechanisms. Alongside a multitude of studies conducted in vitro and in vivo, Monte Carlo computational modelling has been applied in attempts to validate this theory, aiming to identify the chemical interactions that occur along a single particle’s trajectory during FLASH to determine the reaction kinetics and redox chemistry during radiolysis [92, 105, 106]. During conventional fractionated therapy at low dose rates, interactions between incoming ionised particles and intracellular species may occur, but the products of these reactions are unlikely to react with one another due to existing up to minutes apart. Therefore, the chance of ROS produced from each fraction interacting with each other is negligible. Under FLASH conditions, higher dose rates are used that are many orders of magnitude higher than conventional dose rates (> 40 Gy/s vs. 0.03 Gy/s), with ionizing radiation assumed to be evenly distributed almost instantaneously over the entire tumour volume. As such, spurs and tracks interact and overlap at a higher frequency, thereby increasing the initial concentration of e^−^ _eq_, H^*^ and ^*^OH radicals after radiation absorption. These radicals are at a concentration equal to or higher than intracellular O_2_, and as there is a much shorter timeframe between radiation pulses, the chance of radical-radical combination increases. With H_2_O_2_, H_2_O and H_2_ being the major products of these interactions, it is hypothesised that cellular O_2_ is consumed throughout the process, hence explaining why the FLASH effect induces radiolytic oxygen depletion. This also explains why normal tissues are protected during FLASH radiotherapy, as a lack of reactive oxygen species prevents DNA damage in irradiated cells. The duration over which radiation is delivered may also correlate with this. Zlobinskaya presented FLASH instantaneous dose rates of 2 × 10^10^, however irradiation pulses were delivered over 35 min, resulting in a low average dose rate of 0.009 Gy/s [54]. Pulses at ultra-high dose rates, however at a lower frequency and as a result, a longer total irradiation time, may help elucidate mechanisms such as reoxygenation which could occur between pulses if the duration is long enough. Alternative FLASH mechanisms could dominate depending on the total duration of irradiation and instantaneous dose rates used, and not be entirely dependent upon the average dose rate administered.

These predictions outlined by Koch [107] and Spitz et al. [108] lay the foundation for Abolfath et al. [109] and their simulation-based study to test this hypothesis of oxygen depletion. Via analysis of sub-picosecond timescale interactions with Geant4-DNA Monte Carlo modelling, they aimed to perform a molecular dynamics simulation to help explain tissue damage mitigation observed during FLASH irradiation. They simulated damage to a confined portion of DNA irradiated with protons and measured how the molecules surrounding it (including protein, H_2_O and O_2_ molecules) interacted. Conclusions included an increase in ROS with increasing radiation dose, a decrease in ROS with an increase in dose rate, and that under physoxia conditions (4–5% O_2_) the radioprotection of FLASH is maximised. In this instance, Abolfath et al. present that ROS interact through a vast series of hydrogen bonds, preventing ROS diffusion around DNA and reducing the chance of DNA damage. Modelling conducted by Zhu et al. [106] and Zakaria et al. [12] also support these findings. On the contrary, Labarbe et al. [110] present that transient oxygen depletion is not the prime mechanism for the FLASH effect; their model suggesting that a reduction of peroxyl radicals during FLASH is the prime mechanism by which normoxic tissues are protected.

Whilst the majority of simulation work appears to be in support of the oxygen effect and its influence upon particle-FLASH sparing, the translation and application of these results in experimental work is mixed. Beyreuther et al. [69] and their study analysing the effect of 224 MeV proton-FLASH irradiations upon zebrafish morphology depicted minimal differences in pericardial edema and spinal curvature in comparison to CONV treatments. A similar study was conducted by Pawelke et al. [48], instead using 20 MeV electrons, observed reduced incidence of pericardial edema and spinal curvature under low pO_2_ conditions after FLASH irradiations compared to high pO_2_ conditions. This suggests that local oxygen concentration may have an impact upon the effectiveness of FLASH, where high pO_2_ irradiations masked a potential FLASH effect and zebrafish morphology exhibited minimal differences compared to CONV. This also aligns with whole brain irradiation studies in mice, where Montay-Gruel et al. [36] displayed that the neurocognitive benefits of electron-FLASH were lost when brain tissue pO_2_ was artificially doubled via carbogen breathing.

Water phantom studies, such as those conducted by Jansen et al. [111], seem to oppose these conclusions. Contrary to previously mentioned modelling studies, they state that for 10 Gy FLASH irradiations, oxygen consumption decreases with increasing dose rate nonlinearly due to lower steady state values of e^−^ _aq_ radicals, contradicting the apparent sparing effect of FLASH at low pO_2_. Bovine serum albumin irradiations with electron-FLASH conducted by Cao et al. [46] also support these findings (Table 2), although in vivo measurements failed to achieve oxygen depletion under CONV conditions due to reoxygenation from vasculature. However, additional analogous experiments performed in vivo are required to further link oxygen depletion to the FLASH effect and to identify alternative biological explanations. Interestingly, the increased normal tissue sparing effect of FLASH at low pO_2_ levels also appears contradictory to previous claims hypothesising that this effect would be inconsequential in comparable treatments of hypoxic tumourigenic tissues. An in vitro study by Adrian et al. [40] aligns with this, showing a FLASH tissue sparing effect in hypoxic prostate cancer cells. Spitz et al. [108] postulate that normal tissues can limit Fenton type reactions that yield ROS, thereby providing a potential explanation for this discrepancy. Regardless, translation of this sparing effect on in vivo, hypoxic tumourigenic models is yet to be thoroughly explored.

From these experimental data, the consensus appears to be that oxygen depletion does contribute towards healthy tissue sparing, this effect being greater at low pO_2_ levels. Modelling of oxygen kinetics [105] and experiments utilising multicellular spheroids [112] suggest that FLASH depletion has negligible impact upon antitumour efficacy in already highly hypoxic tumour cores. Regardless, these data present multiple avenues for future work in addressing key questions concerning the feasibility of FLASH, limited not only to the oxygen effect but also other unknown biochemical mechanisms of the FLASH effect.

## Conclusion

Particle therapy delivered in a particle-FLASH context appears to hold much potential in discovery of beneficial radiobiological response in both normal tissues and tumours. However, there are several areas which must be understood before this treatment modality can be translated safely in clinical settings. Many current data appear to present experimental artefacts and limitations. A major omission in many publications is a solid reporting on dosimetry and quality control. For ultra-high dose rates, the errors in dose are much more prone to significantly deviate from quoted doses compared to low dose rates. The impacts due to irradiation within the Bragg peak compared to the plateau region also remain to be identified. Key limitations of currently published literature stem from a lack of resolving the precise biological processes and mechanisms responsible for the normal tissue sparing effect, leaving much to be addressed in future preclinical work. Whilst the oxygen depletion hypothesis appears to be a compelling explanation to these underlying questions, there should be more emphasis on experiments which accurately represent in vivo conditions during oxygen depletion that are truly reflective of the tumour microenvironment. Along with this, whilst analysing DSB formation under FLASH irradiation conditions is valuable, more rigorous reporting of beam characteristics, doses administered, and DSB quantification techniques are also required to draw more accurate comparisons of the biological outcomes between experiments. Producing stricter, better-defined characteristics of “FLASH” or “continuous” beams of set dose rates is also of relevance, for instance there is evidence that quasi-continuous beams with a relatively low instantaneous dose rate (5 × 10^4^ Gy/s) may contribute to not observing a FLASH effect [69]. Factors such as pulse structure, time between pulses and instantaneous versus average dose rate further complicate the biological impact of FLASH. Quantifying gene and protein expression under FLASH is also necessary to determine the differential action of DNA repair pathways between cancer and normal cells, as well as determining differences in cell cycle regulation, immune system modulation, and inflammation. Future work focusing on which dose rates, irradiation times, reactive species, and biochemical conditions optimally promote a beneficial particle-FLASH effect will contribute greatly towards progression of this phenomenon into clinical application.

## References

[1] Baskar R, Lee KA, Yeo R, Yeoh K-W (2012). Cancer and Radiation Therapy: current advances and future directions. Int J Med Sci.

[2] Barnett GC, West CML, Dunning AM, Elliott RM, Coles CE, Pharoah PDP (2009). Normal tissue reactions to radiotherapy: towards tailoring treatment dose by genotype. Nat Rev Cancer.

[3] Paganetti H, Beltran C, Both S, Dong L, Flanz J, Furutani K et al (2021) Roadmap: proton therapy physics and biology. Phys Med Biol 66(5). 10.1088/1361-6560/abcd16 10.1088/1361-6560/abcd16 PMC9275016 33227715

[4] Giuranno L, Ient J, De Ruysscher D, Vooijs MA (2019) Radiation-Induced Lung Injury (RILI). Front Oncol 9. 10.3389/fonc.2019.00877 10.3389/fonc.2019.00877 PMC6743286 31555602

[5] Palmer SL, Goloubeva O, Reddick WE, Glass JO, Gajjar A, Kun L (2001). Patterns of Intellectual Development among Survivors of Pediatric Medulloblastoma: a longitudinal analysis. J Clin Oncol.

[6] Mostoufi-Moab S, Grimberg A (2010). Pediatric brain tumor treatment: growth consequences and their management. Pediatr Endocrinol Rev.

[7] Dracham CB, Shankar A, Madan R (2018). Radiation induced secondary malignancies: a review article. Radiation Oncol J.

[8] De Ruysscher D, Niedermann G, Burnet NG, Siva S, Lee AWM, Hegi-Johnson F (2019) Radiotherapy toxicity. Nat Reviews Disease Primers 5(1). 10.1038/s41572-019-0064-5 10.1038/s41572-019-0064-5 30792503

[9] Moreno AC, Frank SJ, Garden AS, Rosenthal DI, Fuller CD, Gunn GB (2019). Intensity modulated proton therapy (IMPT) – the future of IMRT for head and neck cancer. Oral Oncol.

[10] Ohno T (2013) Particle radiotherapy with carbon ion beams. EPMA J 4(1). 10.1186/1878-5085-4-9 10.1186/1878-5085-4-9 PMC3598788 23497542

[11] Soto LA, Casey KM, Wang J, Blaney A, Manjappa R, Breitkreutz D et al (2020) FLASH irradiation results in reduced severe skin toxicity compared to conventional-dose-rate irradiation. Radiat Res 194(6). 10.1667/rade-20-00090 10.1667/RADE-20-00090 PMC7855987 32853385

[12] Zakaria AM, Colangelo NW, Meesungnoen J, Azzam EI, Plourde M-É, Jay-Gerin J-P (2020) Ultra-High Dose-Rate, Pulsed (FLASH) Radiotherapy with Carbon Ions: Generation of early, transient, highly oxygenated Conditions in the Tumor Environment. Radiat Res 194(6). 10.1667/rade-19-00015.1 10.1667/RADE-19-00015.1 PMC7856087 32853343

[13] Zhou G (2020). Mechanisms underlying FLASH radiotherapy, a novel way to enlarge the differential responses to ionizing radiation between normal and tumor tissues. Radiation Med Prot.

[14] Gao F, Yang Y, Zhu H, Wang J, Xiao D, Zhou Z (2022). First demonstration of the FLASH effect with ultrahigh dose rate high-energy X-rays. Radiother Oncol.

[15] Shi X, Yang Y, Zhang W, Wang J, Xiao D, Ren H et al (2022) FLASH X-ray spares intestinal crypts from pyroptosis initiated by cGAS-STING activation upon radioimmunotherapy. Proc Natl Acad Sci 119(43):e2208506119. 10.1073/pnas.2208506119 10.1073/pnas.2208506119 PMC9618056 36256824

[16] Montay-Gruel P, Bouchet A, Jaccard M, Patin D, Serduc R, Aim W (2018). X-rays can trigger the FLASH effect: ultra-high dose-rate synchrotron light source prevents normal brain injury after whole brain irradiation in mice. Radiother Oncol.

[17] Moher D, Liberati A, Tetzlaff J, Altman DG (2009) Preferred reporting items for systematic reviews and Meta-analyses: the PRISMA Statement. PLoS Med 6(7). 10.1371/journal.pmed.1000097 10.1371/journal.pmed.1000097 PMC2707599 19621072

[18] Favaudon V, Caplier L, Monceau V, Pouzoulet F, Sayarath M, Fouillade C et al (2014) Ultrahigh dose-rate FLASH irradiation increases the differential response between normal and tumor tissue in mice. Sci Transl Med 6(245). 10.1126/scitranslmed.3008973 10.1126/scitranslmed.3008973 25031268

[19] Kirby-Smith JS, Dolphin GW (1958). Chromosome breakage at High Radiation Dose-Rates. Nature.

[20] Dewey DL, Boag JW (1959). Modification of the Oxygen Effect when Bacteria are given large pulses of Radiation. Nature.

[21] Sutton HC, Rotblat J (1957). Dose-rate Effects in Radiation-Induced Chemical reactions. Nature.

[22] Epp ER, Weiss H, Santomasso A (1968) The Oxygen Effect in bacterial cells irradiated with high-intensity pulsed Electrons. Radiat Res 34(2). 10.2307/3572557 4869656

[23] Town CD (1967). Effect of high dose rates on survival of mammalian cells. Nature.

[24] Straub JM, New J, Hamilton CD, Lominska C, Shnayder Y, Thomas SM (2015). Radiation-induced fibrosis: mechanisms and implications for therapy. J Cancer Res Clin Oncol.

[25] Kim H, Pyo H, Noh JM, Lee W, Park B, Park HY et al (2019) Preliminary result of definitive radiotherapy in patients with non-small cell lung cancer who have underlying idiopathic pulmonary fibrosis: comparison between X-ray and proton therapy. Radiat Oncol 14(1). 10.1186/s13014-019-1221-4 10.1186/s13014-019-1221-4 PMC6348683 30691496

[26] Hornsey S, Bewley DK (1971). Hypoxia in Mouse Intestine Induced by Electron Irradiation at High Dose-rates. Int J Radiation Biology Relat Stud Phys Chem Med.

[27] Field SB, Bewley DK (1974). Effects of dose-rate on the Radiation response of rat skin. International Journal of Radiation Biology and Related Studies in Physics. Chem Med.

[28] Purrott RJ, Reeder EJ, Lovell S (1977). Chromosome aberration yields Induced in Human Lymphocytes by 15 MeV electrons given at a conventional dose-rate and in Microsecond Pulses. Int J Radiation Biology Relat Stud Phys Chem Med.

[29] Beyreuther E, Karsch L, Laschinsky L, Leßmann E, Naumburger D, Oppelt M (2015). Radiobiological response to ultra-short pulsed megavoltage electron beams of ultra-high pulse dose rate. Int J Radiat Biol.

[30] Laschinsky L, Karsch L, Leßmann E, Oppelt M, Pawelke J, Richter C (2016). Radiobiological influence of megavoltage electron pulses of ultra-high pulse dose rate on normal tissue cells. Radiat Environ Biophys.

[31] Montay-Gruel P, Petersson K, Jaccard M, Boivin G, Germond J-F, Petit B (2017). Irradiation in a flash: unique sparing of memory in mice after whole brain irradiation with dose rates above 100 Gy/s. Radiother Oncol.

[32] Loo BW, Schuler E, Lartey FM, Rafat M, King GJ, Trovati S et al (2017) (P003) Delivery of ultra-rapid flash radiation therapy and demonstration of normal tissue sparing after abdominal irradiation of mice. Int J Radiat Oncol Biol Phys 98(2). 10.1016/j.ijrobp.2017.02.101

[33] Vozenin M-C, De Fornel P, Petersson K, Favaudon V, Jaccard M, Germond J-F (2019). The advantage of FLASH Radiotherapy confirmed in mini-pig and cat-cancer patients. Clin Cancer Res.

[34] Bourhis J, Sozzi WJ, Jorge PG, Gaide O, Bailat C, Duclos F (2019). Treatment of a first patient with FLASH-radiotherapy. Radiother Oncol.

[35] Venkatesulu BP, Sharma A, Pollard-Larkin JM, Sadagopan R, Symons J, Neri S et al (2019) Ultra high dose rate (35 Gy/sec) radiation does not spare the normal tissue in cardiac and splenic models of lymphopenia and gastrointestinal syndrome. Sci Rep 9(1). 10.1038/s41598-019-53562-y 10.1038/s41598-019-53562-y PMC6868225 31748640

[36] Montay-Gruel P, Acharya MM, Petersson K, Alikhani L, Yakkala C, Allen BD et al (2019) Long-term neurocognitive benefits of FLASH radiotherapy driven by reduced reactive oxygen species. Proc Natl Acad Sci 116(22):10943–10951. 10.1073/pnas.1901777116 10.1073/pnas.1901777116 PMC6561167 31097580

[37] Simmons DA, Lartey FM, Schüler E, Rafat M, King G, Kim A (2019). Reduced cognitive deficits after FLASH irradiation of whole mouse brain are associated with less hippocampal dendritic spine loss and neuroinflammation. Radiother Oncol.

[38] Alaghband Y, Cheeks SN, Allen BD, Montay-Gruel P, Doan N-L, Petit B et al (2020) Neuroprotection of Radiosensitive Juvenile mice by Ultra-High Dose Rate FLASH Irradiation. Cancers 12(6). 10.3390/cancers12061671 10.3390/cancers12061671 PMC7352849 32599789

[39] Montay-Gruel P, Markarian M, Allen BD, Baddour JD, Giedzinski E, Jorge PG et al (2020) Ultra-High-Dose-Rate FLASH irradiation limits reactive gliosis in the brain. Radiat Res 194(6). 10.1667/rade-20-00067.1 10.1667/RADE-20-00067.1 PMC7856066 32853387

[40] Adrian G, Konradsson E, Lempart M, Bäck S, Ceberg C, Petersson K (2020) The FLASH effect depends on oxygen concentration. Br J Radiol 93(1106). 10.1259/bjr.20190702 10.1259/bjr.20190702 PMC7055454 31825653

[41] Allen BD, Acharya MM, Montay-Gruel P, Jorge PG, Bailat C, Petit B (2020). Maintenance of tight Junction Integrity in the absence of vascular dilation in the brain of mice exposed to Ultra-High-Dose-Rate FLASH Irradiation. Radiat Res.

[42] Breitkreutz DY, Shumail M, Bush KK, Tantawi SG, Maxime PG, Loo BW (2020) Initial steps towards a clinical FLASH Radiotherapy System: Pediatric Whole Brain irradiation with 40 MeV electrons at FLASH Dose Rates. Radiat Res 194(6). 10.1667/rade-20-00069.1 10.1667/RADE-20-00069.1 PMC7856241 32991725

[43] Levy K, Natarajan S, Wang J, Chow S, Eggold JT, Loo PE et al (2020) Abdominal FLASH irradiation reduces radiation-induced gastrointestinal toxicity for the treatment of ovarian cancer in mice. Sci Rep 10(1). 10.1038/s41598-020-78017-7 10.1038/s41598-020-78017-7 PMC7728763 33303827

[44] Fouillade C, Curras-Alonso S, Giuranno L, Quelennec E, Heinrich S, Bonnet-Boissinot S (2020). FLASH irradiation spares lung progenitor cells and limits the incidence of radio-induced senescence. Clin Cancer Res.

[45] Chabi S, To THV, Leavitt R, Poglio S, Jorge PG, Jaccard M (2021). Ultra-high-dose-rate FLASH and conventional-dose-rate irradiation differentially affect human acute lymphoblastic leukemia and normal hematopoiesis. Int J Radiation Oncology*Biology*Physics.

[46] Cao X, Zhang R, Esipova TV, Allu SR, Ashraf R, Rahman M (2021). Quantification of Oxygen Depletion during FLASH Irradiation in Vitro and in vivo. Int J Radiation Oncology*Biology*Physics.

[47] Montay-Gruel P, Acharya MM, Gonçalves Jorge P, Petit B, Petridis IG, Fuchs P (2021). Hypofractionated FLASH-RT as an effective treatment against Glioblastoma that reduces Neurocognitive Side Effects in mice. Clin Cancer Res.

[48] Pawelke J, Brand M, Hans S, Hideghéty K, Karsch L, Lessmann E (2021). Electron dose rate and oxygen depletion protect zebrafish embryos from radiation damage. Radiother Oncol.

[49] Gasymova E, Meier V, Guscetti F, Cancedda S, Roos M, Rohrer Bley C (2017) Retrospective clinical study on outcome in cats with nasal planum squamous cell carcinoma treated with an accelerated radiation protocol. BMC Vet Res 13(1). 10.1186/s12917-017-1018-3 10.1186/s12917-017-1018-3 PMC5381142 28376918

[50] Konradsson E, Arendt ML, Bastholm Jensen K, Børresen B, Hansen AE, Bäck S et al (2021) Establishment and initial experience of clinical FLASH Radiotherapy in Canine Cancer Patients. Front Oncol 11. 10.3389/fonc.2021.658004 10.3389/fonc.2021.658004 PMC8155542 34055624

[51] Wei S, Lin H, Choi JI, Simone CB, Kang M (2021) A Novel Proton Pencil Beam scanning FLASH RT Delivery Method enables optimal OAR sparing and Ultra-High Dose Rate Delivery: a Comprehensive Dosimetry Study for Lung Tumors. Cancers 13(22). 10.3390/cancers13225790 10.3390/cancers13225790 PMC8616118 34830946

[52] Kim MM, Verginadis II, Goia D, Haertter A, Shoniyozov K, Zou W et al (2021) Comparison of FLASH Proton Entrance and the spread-out Bragg Peak Dose Regions in the sparing of mouse intestinal crypts and in a pancreatic tumor model. Cancers 13(16). 10.3390/cancers13164244 10.3390/cancers13164244 PMC8392865 34439398

[53] Evans T, Cooley J, Wagner M, Yu T, Zwart T (2021). Demonstration of the FLASH Effect within the spread-out Bragg Peak after Abdominal Irradiation of mice. Int J Part Therapy.

[54] Zlobinskaya O, Siebenwirth C, Greubel C, Hable V, Hertenberger R, Humble N (2014). The Effects of Ultra-High Dose Rate Proton Irradiation on Growth Delay in the treatment of human tumor xenografts in Nude mice. Radiat Res.

[55] TASHIRO M, YOSHIDA Y, OIKE T, NAKAO M, YUSA K, HIROTA Y (2022). First human cell experiments with FLASH Carbon Ions. Anticancer Res.

[56] Guo Z, Buonanno M, Harken A, Zhou G, Hei TK (2022). Mitochondrial damage response and fate of normal cells exposed to FLASH Irradiation with Protons. Radiat Res.

[57] Dokic I, Meister S, Bojcevski J, Tessonnier T, Walsh D, Knoll M (2022). Neuroprotective Effects of Ultra-High Dose Rate FLASH Bragg Peak Proton Irradiation. Int J Radiat Oncol Biol Phys.

[58] Manti L, Perozziello FM, Borghesi M, Candiano G, Chaudhary P, Cirrone GAP (2017). The radiobiology of laser-driven particle beams: focus on sub-lethal responses of normal human cells. J Instrum.

[59] Doria D, Kakolee KF, Kar S, Litt SK, Fiorini F, Ahmed H et al (2012) Biological effectiveness on live cells of laser driven protons at dose rates exceeding 109Gy/s. AIP Adv 2(1). 10.1063/1.3699063

[60] Schmid TE, Dollinger G, Hable V, Greubel C, Zlobinskaya O, Michalski D (2011). The effectiveness of 20 MeV protons at Nanosecond Pulse Lengths in Producing chromosome aberrations in human-Hamster hybrid cells. Radiat Res.

[61] Auer S, Hable V, Greubel C, Drexler GA, Schmid TE, Belka C et al (2011) Survival of tumor cells after proton irradiation with ultra-high dose rates. Radiat Oncol 6(1). 10.1186/1748-717x-6-139 10.1186/1748-717X-6-139 PMC3215966 22008289

[62] Schmid TE, Dollinger G, Hauptner A, Hable V, Greubel C, Auer S (2009). No evidence for a different RBE between Pulsed and continuous 20 MeV protons. Radiat Res.

[63] Buonanno M, Grilj V, Brenner DJ (2019). Biological effects in normal cells exposed to FLASH dose rate protons. Radiother Oncol.

[64] Rogakou EP, Pilch DR, Orr AH, Ivanova VS, Bonner WM (1998). DNA double-stranded breaks induce histone H2AX phosphorylation on serine 139. J Biol Chem.

[65] Bayart E, Flacco A, Delmas O, Pommarel L, Levy D, Cavallone M et al (2019) Fast dose fractionation using ultra-short laser accelerated proton pulses can increase cancer cell mortality, which relies on functional PARP1 protein. Sci Rep 9(1). 10.1038/s41598-019-46512-1 10.1038/s41598-019-46512-1 PMC6626007 31300704

[66] Raschke S, Spickermann S, Toncian T, Swantusch M, Boeker J, Giesen U et al (2016) Ultra-short laser-accelerated proton pulses have similar DNA-damaging effectiveness but produce less immediate nitroxidative stress than conventional proton beams. Sci Rep 6(1). 10.1038/srep32441 10.1038/srep32441 PMC5006042 27578260

[67] Zlobinskaya O, Dollinger G, Michalski D, Hable V, Greubel C, Du G (2012). Induction and repair of DNA double-strand breaks assessed by gamma-H2AX foci after irradiation with pulsed or continuous proton beams. Radiat Environ Biophys.

[68] Hanton F, Chaudhary P, Doria D, Gwynne D, Maiorino C, Scullion C et al (2019) DNA DSB Repair Dynamics following irradiation with laser-driven protons at Ultra-High Dose Rates. Sci Rep 9(1). 10.1038/s41598-019-40339-6 10.1038/s41598-019-40339-6 PMC6418121 30872656

[69] Beyreuther E, Brand M, Hans S, Hideghéty K, Karsch L, Leßmann E (2019). Feasibility of proton FLASH effect tested by zebrafish embryo irradiation. Radiother Oncol.

[70] Rama N, Saha T, Shukla S, Goda C, Milewski D, Mascia AE (2019). Improved tumor control through T-cell infiltration modulated by ultra-high dose rate proton FLASH using a clinical pencil beam scanning proton system. Int J Radiat Oncol Biol Phys.

[71] Abel E, Girdhani S, Jackson I, Eley J, Katsis A, Marshall A (2019). Characterization of radiation-induced lung fibrosis and mode of cell death using single and multi-pulsed proton flash irradiation. Int J Radiat Oncol Biol Phys.

[72] Diffenderfer ES, Verginadis II, Kim MM, Shoniyozov K, Velalopoulou A, Goia D (2020). Design, implementation, and in vivo validation of a novel proton FLASH radiation therapy system. Int J Radiat Oncol Biol Phys.

[73] Zhang Q, Cascio E, Li C, Yang Q, Gerweck LE, Huang P et al (2020) FLASH investigations using protons: design of Delivery System, preclinical setup and confirmation of FLASH Effect with Protons in Animal Systems. Radiat Res 194(6). 10.1667/rade-20-00068.1 10.1667/RADE-20-00068.1 32991708

[74] Cunningham S, McCauley S, Vairamani K, Speth J, Girdhani S, Abel E et al (2021) FLASH Proton Pencil Beam scanning irradiation minimizes Radiation-Induced Leg contracture and skin toxicity in mice. Cancers 13(5). 10.3390/cancers13051012 10.3390/cancers13051012 PMC7957631 33804336

[75] Velalopoulou A, Karagounis IV, Cramer GM, Kim MM, Skoufos G, Goia D (2021). FLASH Proton Radiotherapy spares normal epithelial and mesenchymal tissues while preserving Sarcoma Response. Cancer Res.

[76] Tinganelli W, Sokol O, Quartieri M, Puspitasari A, Dokic I, Abdollahi A (2022). Ultra-high dose rate (FLASH) carbon ion irradiation: Dosimetry and first cell experiments. Int J Radiat Oncol Biol Phys.

[77] Singers Sørensen B, Krzysztof Sitarz M, Ankjærgaard C, Johansen J, Andersen CE, Kanouta E (2022). In vivo validation and tissue sparing factor for acute damage of pencil beam scanning proton FLASH. Radiother Oncol.

[78] Karsch L, Pawelke J, Brand M, Hans S, Hideghéty K, Jansen J (2022). Beam pulse structure and dose rate as determinants for the flash effect observed in zebrafish embryo. Radiother Oncol.

[79] Tinganelli W, Weber U, Puspitasari A, Simoniello P, Abdollahi A, Oppermann J (2022). FLASH with carbon ions: tumor control, normal tissue sparing, and distal metastasis in a mouse osteosarcoma model. Radiother Oncol.

[80] Sørensen BS, Sitarz MK, Ankjærgaard C, Johansen JG, Andersen CE, Kanouta E (2022). Pencil beam scanning proton FLASH maintains tumor control while normal tissue damage is reduced in a mouse model. Radiother Oncol.

[81] Kacem H, Psoroulas S, Boivin G, Folkerts M, Grilj V, Lomax T (2022). Comparing radiolytic production of H < sub > 2 O < sub > 2 and development of zebrafish embryos after ultra high dose rate exposure with electron and transmission proton beams. Radiother Oncol.

[82] Bin J, Obst-Huebl L, Mao J-H, Nakamura K, Geulig LD, Chang H (2022). A new platform for ultra-high dose rate radiobiological research using the BELLA PW laser proton beamline. Sci Rep.

[83] Varian, aSHC (2022) : Feasibility Study of FLASH Radiotherapy for the Treatment of Symptomatic Bone Metastases. https://ClinicalTrials.gov/show/NCT04592887 Accessed

[84] Chow R, Kang M, Wei S, Choi JI, Press RH, Hasan S (2021). FLASH radiation therapy: review of the literature and considerations for future research and proton therapy FLASH trials. Appl Radiat Oncol.

[85] Mascia AE, Daugherty EC, Zhang Y, Lee E, Xiao Z, Sertorio M (2022). Proton FLASH Radiotherapy for the treatment of symptomatic bone metastases: the FAST-01 Nonrandomized Trial. JAMA Oncol.

[86] Zakaria AM, Colangelo NW, Meesungnoen J, Jay-Gerin J-P (2021). Transient hypoxia in water irradiated by swift carbon ions at ultra-high dose rates: implication for FLASH carbon-ion therapy. Can J Chem.

[87] Wilson JD, Hammond EM, Higgins GS, Petersson K (2020) Ultra-High Dose Rate (FLASH) Radiotherapy: silver bullet or Fool’s gold? Front Oncol 9. 10.3389/fonc.2019.01563 10.3389/fonc.2019.01563 PMC6979639 32010633

[88] Alizadeh E, Sanz AG, García G, Sanche L (2013). Radiation damage to DNA: the Indirect Effect of Low-Energy Electrons. J Phys Chem Lett.

[89] Srinivas US, Tan BWQ, Vellayappan BA, Jeyasekharan AD (2019) ROS and the DNA damage response in cancer. Redox Biol 25. 10.1016/j.redox.2018.101084 10.1016/j.redox.2018.101084 PMC6859528 30612957

[90] Maier P, Hartmann L, Wenz F, Herskind C (2016) Cellular Pathways in response to Ionizing Radiation and their targetability for Tumor Radiosensitization. Int J Mol Sci 17(1). 10.3390/ijms17010102 10.3390/ijms17010102 PMC4730344 26784176

[91] Durante M, Vozenin M-C, Breneman J, Dong L, Scifoni E (2021). Panel discussion – FLASH therapy.

[92] Boscolo D, Scifoni E, Durante M, Kramer M, Fuss MC (2021). May oxygen depletion explain the FLASH effect? A chemical track structure analysis. Radiother Oncol.

[93] Kim Y-E, Gwak S-H, Hong B-J, Oh J-M, Choi H-S, Kim MS (2021). Effects of Ultra-high doserate FLASH irradiation on the Tumor Microenvironment in Lewis Lung Carcinoma: role of myosin light chain. Int J Radiation Oncology*Biology*Physics.

[94] Ilicic K, Combs SE, Schmid TE (2018) New insights in the relative radiobiological effectiveness of proton irradiation. Radiat Oncol 13(1). 10.1186/s13014-018-0954-9 10.1186/s13014-018-0954-9 PMC5771069 29338744

[95] Nickoloff JA, Sharma N, Taylor L, Clustered DNA, Double-Strand (2020) Breaks: Biological Effects and Relevance to Cancer Radiotherapy. Genes 11(1). 10.3390/genes11010099 10.3390/genes11010099 PMC7017136 31952359

[96] Alhmoud JF, Woolley JF, Al Moustafa A-E, Malki MI (2020) DNA Damage/Repair management in cancers. Cancers 12(4). 10.3390/cancers12041050 10.3390/cancers12041050 PMC7226105 32340362

[97] Szymonowicz K, Krysztofiak A, van der Linden J, Kern A, Deycmar S, Oeck S et al (2020) Proton Irradiation increases the necessity for homologous recombination repair along with the indispensability of non-homologous end joining. Cells 9(4). 10.3390/cells9040889 10.3390/cells9040889 PMC7226794 32260562

[98] Konopacka M, Rogoliński J, Sochanik A, Ślosarek K (2016). Can high dose rates used in cancer radiotherapy change therapeutic effectiveness?. Współczesna Onkologia.

[99] Jeong H, Bok S, Hong B-J, Choi H-S, Ahn GO (2016) Radiation-induced immune responses: mechanisms and therapeutic perspectives. Blood Res 51(3). 10.5045/br.2016.51.3.157 10.5045/br.2016.51.3.157 PMC5054246 27722125

[100] Takahashi J, Nagasawa S (2020) Immunostimulatory Effects of Radiotherapy for local and systemic control of Melanoma: a review. Int J Mol Sci 21(23). 10.3390/ijms21239324 10.3390/ijms21239324 PMC7730562 33297519

[101] Demaria S, Formenti SC (2016) Can abscopal effects of local radiotherapy be predicted by modeling T cell trafficking? J Immunother Cancer 4(1). 10.1186/s40425-016-0133-1 10.1186/s40425-016-0133-1 PMC4869282 27190630

[102] Jin JY, Gu A, Wang W, Oleinick NL, Machtay M, Kong FM (2020) FLASH dose rate effect on circulating immune cells: a potential mechanism for FLASH-RT? Int J Radiat Oncol Biol Phys 108(3). 10.1016/j.ijrobp.2020.07.2079 10.1016/j.radonc.2020.04.054 PMC7442672 32387486

[103] Pratx G, Kapp DS (2019). A computational model of radiolytic oxygen depletion during FLASH irradiation and its effect on the oxygen enhancement ratio. Phys Med Biol.

[104] Chaudhary P, Milluzzo G, Ahmed H, Odlozilik B, McMurray A, Prise KM et al (2021) Radiobiology experiments with Ultra-high Dose Rate Laser-Driven Protons: Methodology and State-of-the-art. Front Phys 9. 10.3389/fphy.2021.624963

[105] Petersson K, Adrian G, Butterworth K, McMahon SJ (2020). A quantitative analysis of the role of oxygen tension in FLASH radiation therapy. Int J Radiat Oncol Biol Phys.

[106] Zhu H, Li J, Deng X, Qiu R, Wu Z, Zhang H (2021) Modeling of cellular response after FLASH irradiation: a quantitative analysis based on the radiolytic oxygen depletion hypothesis. Phys Med Biol 66(18). 10.1088/1361-6560/ac226d 10.1088/1361-6560/ac226d 34464946

[107] Koch CJ, Re (2019). Differential impact of FLASH versus conventional dose rate irradiation: Spitz. Radiother Oncol.

[108] Spitz DR, Buettner GR, Petronek MS, St-Aubin JJ, Flynn RT, Waldron TJ (2019). An integrated physico-chemical approach for explaining the differential impact of FLASH versus conventional dose rate irradiation on cancer and normal tissue responses. Radiother Oncol.

[109] Abolfath R, Grosshans D, Mohan R (2020). Oxygen depletion in FLASH ultra-high‐dose‐rate radiotherapy: a molecular dynamics simulation. Med Phys.

[110] Labarbe R, Hotoiu L, Barbier J, Favaudon V (2020). A physicochemical model of reaction kinetics supports peroxyl radical recombination as the main determinant of the FLASH effect. Radiother Oncol.

[111] Jansen J, Knoll J, Beyreuther E, Pawelke J, Skuza R, Hanley R (2021). Does FLASH deplete oxygen? Experimental evaluation for photons, protons, and carbon ions. Med Phys.

[112] Khan S, Bassenne M, Wang J, Manjappa R, Melemenidis S, Breitkreutz DY (2021). Multicellular spheroids as in vitro models of oxygen depletion during FLASH irradiation. Int J Radiat Oncol Biol Phys.

